# Aerobic Training Protects Cardiac Function During Advancing Age: A Meta-Analysis of Four Decades of Controlled Studies

**DOI:** 10.1007/s40279-018-1004-3

**Published:** 2018-10-29

**Authors:** Alexander J. Beaumont, Fergal M. Grace, Joanna C. Richards, Amy K. Campbell, Nicholas F. Sculthorpe

**Affiliations:** 1 School of Health and Life Science, Institute of Clinical Exercise and Health Science, University of the West of Scotland, Glasgow, UK; 2 Human Movement and Sports Science Group, Faculty of Health, Federation University Australia, Ballarat, VIC Australia; 3 School of Sport Science and Physical Activity, Institute of Sport and Physical Activity Research, University of Bedfordshire, Bedford, UK

## Abstract

**Background:**

In contrast to younger athletes, there is comparatively less literature examining cardiac structure and function in older athletes. However, a progressive accumulation of studies during the past four decades offers a body of literature worthy of systematic scrutiny.

**Objectives:**

We conducted a systematic review, meta-analysis and meta-regression of controlled echocardiography studies comparing left ventricular (LV) structure and function in aerobically trained older athletes (> 45 years) with age-matched untrained controls, in addition to investigating the influence of chronological age.

**Methods:**

Electronic databases were searched from inception to January 2018 before conducting a random-effects meta-analysis to calculate pooled differences in means, effect size and 95% confidence intervals (CIs). Study heterogeneity was reported using Cochran’s *Q* and *I*^2^ statistic.

**Results:**

Overall, 32 studies (644 athletes; 582 controls) were included. Athletes had greater LV end-diastolic diameter (3.65 mm, 95% CI 2.66–4.64), interventricular septal thickness (1.23 mm, 95% CI 0.85–1.60), posterior wall thickness (1.20 mm, 95% CI 0.83–1.56), LV mass (72 g, 95% CI 46–98), LV mass index (28.17 g·m^2^, 95% CI 19.84–36.49) and stroke volume (13.59 mL, 95% CI 7.20–19.98) (all *p* < 0.01). Athletes had superior global diastolic function [ratio of early (*E*) to late (*A*) mitral inflow velocity (*E*/*A*) 0.18, 95% CI 0.13–0.24, *p* < 0.01; ratio of early (*e*′) to late (*a*′) diastolic annular tissue velocity (*e*′/*a*′) 0.23, 95% CI 0.06–0.40, *p* = 0.01], lower *A* (−8.20 cm·s^−1^, 95% CI −11.90 to −4.51, *p* < 0.01) and *a*′ (−0.72 cm·s^−1^, 95% CI −1.31 to −0.12, *p* = 0.02), and more rapid *e*′ (0.96 cm·s^−1^, 95% CI 0.05–1.86, *p* = 0.04). Meta-regression for chronological age identified that athlete–control differences, in the main, are maintained during advancing age.

**Conclusions:**

Athletic older men have larger cardiac dimensions and enjoy more favourable cardiac function than healthy, non-athletic counterparts. Notably, the athlete groups maintain these effects during chronological ageing.

**Electronic supplementary material:**

The online version of this article (10.1007/s40279-018-1004-3) contains supplementary material, which is available to authorized users.

## Key Points

**Table:** 

Trained older men have larger left ventricular morphology and superior diastolic function than age-matched untrained yet healthy controls, determined by conventional echocardiography.
The functional adaptations noted in older athletes are, in the main, maintained with chronological age from middle and into older age.
Aerobic exercise is an effective non-pharmacological therapy to preserve cardiac function during ageing and is maintained with continuous exercise therapy.

## Introduction

It is well established that a lifestyle consisting of regular physical exertion is associated with reduced cardiovascular risk and all-cause mortality across the age spectrum [1, 2]. Despite this, epidemiological studies consistently identify older adults as the least physically active demographic where few achieve the recommended levels of physical activity required to accrue health benefits [3]. Studies of ageing athletes observe relatively high levels of cardiovascular reserve (maximal aerobic capacity) compared with their sedentary counterparts [4]. In this respect, the ‘masters athlete’ model offers a unique non-pharmacological model to differentiate the inexorable from the preventable mechanisms of cardiovascular ageing. In studies of cardiovascular function, endurance-trained masters athletes have superior functional capacity and cardiovascular reserve to their sedentary peers, and are comparable in these respects to non-athletes who are years (and often decades) their junior. As this is not the focus of the present review, we direct readers to the eminent works from Professor Benjamin Levine (University of Texas) and Professor Douglas R. Seals (University of Colorado) [5, 6].

A major limitation of this area, however, is that randomised controlled trials of life-long exercise are highly unlikely, and therefore researchers must rely on evidence from animal models and controlled observational studies to further our understanding of the effects of age on cardiac structure and function [5, 7–9]. Indeed, the pleiotropic benefits of aerobic endurance exercise have been acknowledged as a potential non-pharmacological mitigant to deterioration of left ventricular (LV) diastolic function [10]. Although this body of evidence is encouraging, it is often hampered by small sample sizes in controlled observational studies which suffer from low statistical power, methodological inconsistencies and untested external validity. In contrast to a wealth of data in younger cohorts < 45 years, including several meta-analyses of LV, right ventricular (RV) and left atrial (LA) structure and function in both male [11–17] and female [14, 17, 18] athletes, there have been narrative reviews [10, 19–21] but no systematic synthesis of evidence in older athletes relative to matched, untrained controls. Further, in the absence of prospective investigations of multiple decades in duration, quantifying the influence of chronological age on exercise-related morphological and functional adaptations must be realised through cross-sectional studies, across the age spectrum from middle to older age (i.e. > 45 years).

In light of this, we undertook a systematic review and meta-analysis of studies using echocardiography to (1) compare LV morphology and systolic and diastolic function in older athletes versus age-matched sedentary but otherwise healthy controls and (2) employ meta-regression to explore the influence of chronological age on cardiac structure and function in the ageing athlete.

## Methods

The systematic search processes, evaluation, analysis, and reporting were conducted and presented in accordance with the Preferred Reporting Items for Systematic review and Meta-Analyses (PRISMA) guidelines for reporting systematic reviews and meta-analyses [22].

### Information Sources and Search Strategy

An electronic database search was designed by two authors (AB and NS), who conducted an independent literature search of PubMed (title and abstract), MEDLINE (title) and ScienceDirect (title, abstract and keywords) for published, English-language journal articles from inception to 11 January 2018 (see Electronic Supplementary Material Appendix S1 for the PubMed database search).

### Inclusion Criteria

Studies were eligible for quantitative analysis when meeting the following criteria: (1) study participants were male; (2) mean participant age ≥ 45 years; (3) aged-matched control group; (4) athlete groups were aerobic/endurance trained; (5) control groups were untrained; (6) participants were reported as free from cardiovascular diseases; (7) the study was observational in design and data were recorded at a single time point (including recruitment data from intervention studies); (8) studies used echocardiography; and (9) studies assessed cardiac strain using two-dimensional speckle tracking echocardiography (STE).

We elected to include only endurance athletes participating in predominantly dynamic aerobic activity (for example, distance running, cycling, and rowing). Where study information was unclear, corresponding authors were contacted by email.

### Study Selection and Data Extraction

Following an initial literature search (AB), study data were independently extracted by AB and AC and cross-checked by an arbitrator (NS). Extracted data were entered into a spreadsheet (Microsoft^®^ Excel 2016, Microsoft Corporation, Redmond, WA, USA). Twenty-one measures of LV, RV and LA structure and function were recorded. Cardiac structure measures included (1) interventricular septum (IVS) thickness, (2) LV posterior wall thickness (PWT), (3) left ventricular end-diastolic diameter (LVEDD), (4) relative wall thickness (RWT), (5) left ventricular mass (LVM), (6) LVM index (LVMi), (7) left ventricular end-diastolic volume (LVEDV), (8) right ventricular end-diastolic diameter (RVEDD), and (9) left atrial diameter (LAD). LV systolic function measures by conventional echocardiography were (10) ejection fraction (EF), (11) fractional shortening (FS), (12) stroke volume (SV), (13) systolic annular tissue velocity (*s*′), and by two-dimensional STE, (14) global longitudinal strain (GLS). Diastolic function measures were (15) early mitral inflow velocity (*E*), (16) late mitral inflow velocity (*A*), (17) ratio of early to late mitral inflow velocity (*E*/*A*), (18) early diastolic annular tissue velocity (*e*′), (19) late diastolic annular tissue velocity (*a*′), (20) ratio of early to late diastolic annular tissue velocity (*e*′/*a*′), and (21) ratio of early mitral inflow velocity to early diastolic annular tissue velocity (*E*/*e*′).

Multiple segment reports for mitral annular tissue velocities (septal, lateral wall, inferior wall, anterior wall) were combined to obtain a global value. We included studies employing either pulsed-wave Doppler or colour Doppler techniques to obtain tissue velocities. Data for RWT included both descriptions of RWT and thickness/radius ratio. We elected to only include studies that scaled LVM to body surface area (BSA) in line with current echocardiographic recommendations [23]. GLS was deemed as a global value from the average of multiple segments, and *E*/*e*′ was considered to be an estimate of LV filling pressure [24].

When unsuccessful attempts were made to contact authors, data were extracted from study figures. Study means ± standard deviation (SD) were recorded for all variables; where studies reported the standard error of the mean (SEM), we applied a manual conversion using the formula SD = SEM × √*N*, where *N* is the number of participants. Likewise, when median and range were reported, a manual conversion was applied to convert data into mean and SD, in accordance with the study sample size [25]. For each study, the mean age of athlete and control groups were averaged to obtain a pooled mean. Study quality was assessed for each individual study using a 17-point checklist (Electronic Supplementary Material Table S1) adapted for specificity for this meta-analysis from a previously published checklist used in a similar study of young athletes [13].

### Statistical Analyses

Meta-analyses were executed using Comprehensive Meta-Analysis (Biostat, V 2.2.064, Englewood, NJ, USA). Pooled data using a random-effects model were used to investigate athlete–control differences. Differences in means were calculated for each individual study, and a summary of overall difference in means recorded for all variables. Differences in means in a positive direction indicated greater magnitude of LV structure or function in athletes, with negative direction favouring a greater magnitude in controls. Heterogeneity was reported using Cochran’s *Q* and *I*^2^ statistic and classed as either low, moderate, or high at 25%, 50%, and 75%, respectively [26]. Using pooled athlete and control age (continuous moderator variable), we conducted random-effects (method of moments) meta-regression analysis to examine the relationship between differences in means with chronological age. Meta-regression analysis was performed where there were ten or more studies [27]. Publication bias was addressed using Egger’s regression intercept [28] to test for asymmetry and interpreted conservatively [27]. Statistical significance was granted at *p* ≤ 0.05.

## Results

### Search Outcome

**Fig. 1:**
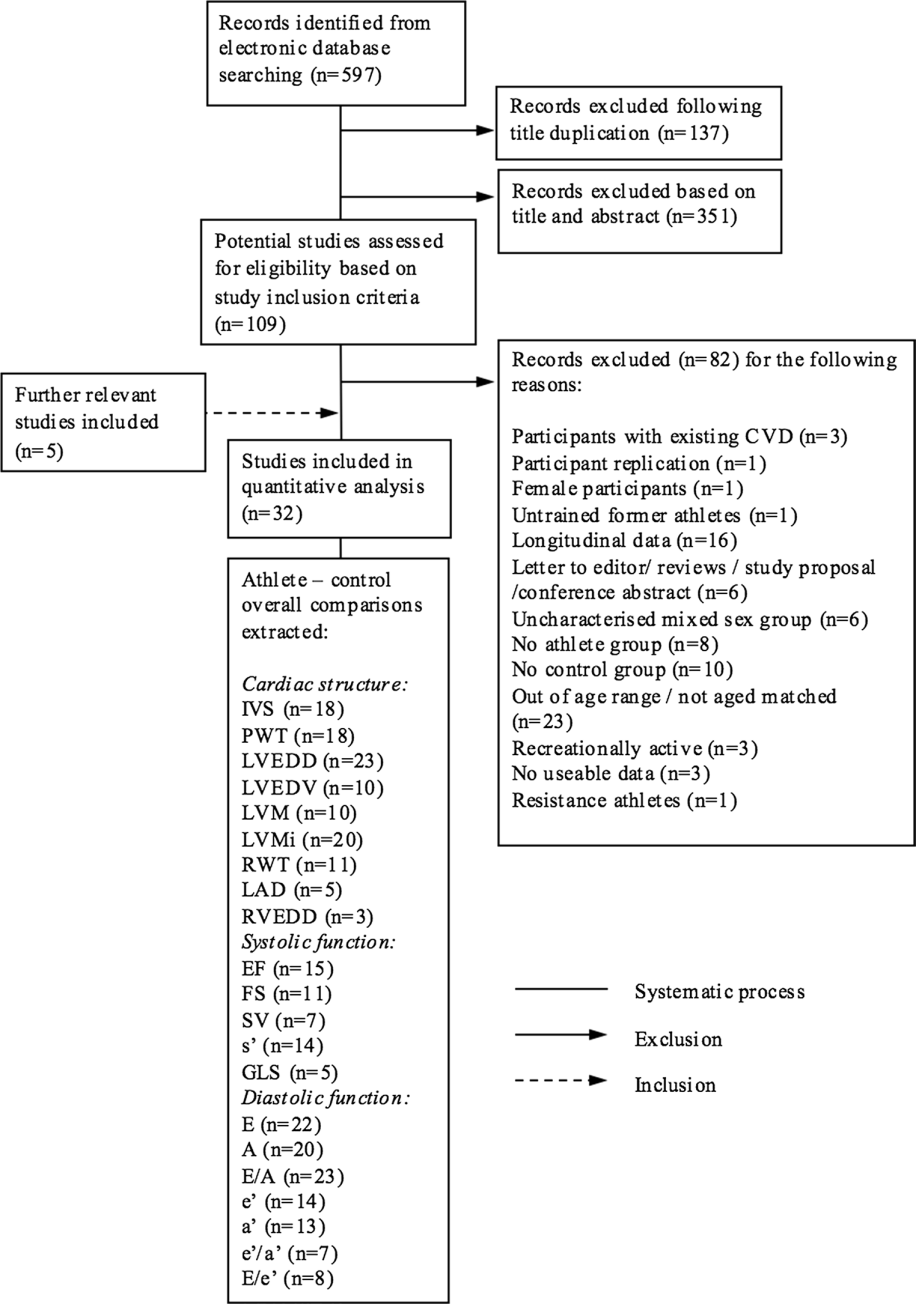
Schematic of systematic process used for identification of eligible studies. Flow diagram of identified records and the filtration process of article inclusion and exclusion. *A* late diastolic mitral inflow velocity, *a*′ late diastolic tissue velocity, *CVD* cardiovascular disease, *E* early diastolic mitral inflow velocity, *e*′ early diastolic tissue velocity, *e*′*/a*′ ratio of early to late diastolic tissue velocity, *E/A* ratio of early to late mitral inflow velocity, *E/e*′ ratio of early diastolic mitral inflow velocity to early diastolic tissue velocity, *EF* ejection fraction, *FS* fractional shortening, *GLS* global longitudinal strain, *IVS* interventricular septal, *LAD* left atrial diameter, *LVEDD* left ventricular end-diastolic diameter, *LVEDV* left ventricular end-diastolic volume, *LVM* left ventricular mass, *LVMi* left ventricular mass index, *PWT* posterior wall thickness, *RVEDD* right ventricular end-diastolic diameter, *RWT* relative wall thickness, *s*′ systolic tissue velocity, *SV* stroke volume

Figure 1 illustrates the systematic filtration process. The electronic database search resulted in 597 records, which were exported to referencing software (Zotero, Fairfax, VA, USA) to manage the systematic process.

Thirty-two studies including 1226 participants [644 athletes (59 ± 8 years) and 582 controls (59 ± 8 years); mean age range 46–74 years] met the inclusion criteria for quantitative analyses [7, 29–59]. This allowed for the following comparisons [number of studies presented in parentheses; study names for each comparison can be found in supplementary forest plots (Electronic Supplementary Material Figs. S1–S21)]: IVS (*n* = 18), PWT (*n* = 18), LVEDD (*n* = 23), LVM (*n* = 10), LVMi (*n* = 20), RWT (*n* = 11), LVEDV (*n* = 10), RVEDD (*n* = 3), LAD (*n* = 5), EF (*n* = 15), FS (*n* = 11), SV (*n* = 7), *s*′ (*n* = 14), GLS (*n* = 5), *E* (*n* = 22), *A* (*n* = 20), *E*/*A* (*n* = 23), *e*′ (*n* = 14), *a*′ (*n* = 13), *e*′/*a*′ (*n* = 7), *E*/*e*′ (*n* = 8) (Fig. 1).

Summarised participant anthropometrics, systolic and diastolic blood pressure and heart rate are presented in Table 1. Characteristics of athlete training regimes and control activity levels are reported in Table 2. LV, RV and LA structure and systolic and diastolic function data for control and athlete groups are presented in Tables 3, 4, and 5, respectively. Table 6 describes the meta-analyses data for athlete–control comparisons including between-study heterogeneity. Electronic Supplementary Material Figs. S1–S36 present forest plots of athlete–control comparisons and meta-regression figures of the athlete–control differences moderated for chronological age.

**Table 1:** Summary of studies and anthropometric data

References	Study group (*n*)	Age (years)	Height (cm)	Mass (kg)	BMI (kg·m^2^)	BSA (m^2^)	HR (beats·min^−1^)	SBP (mmHg)	DBP (mmHg)	Study quality
Child et al. [33]	Control (12)	56.3 ± 7.8	–	–	–	1.93 ± 0.08	62 ± 11	–	–	12
	Runners (9)	53.7 ± 10.6	–	–	–	1.78 ± 0.13	49 ± 7	–	–	
D’Andrea et al. [35]	Control (25)	47.4 ± 2.2	–	–	–	1.88 ± 0.4	73 ± 8	126 ± 6	85 ± 3	14
	Swimmers (40)	48.2 ± 3.4	–	–	–	1.89 ± 0.5	50 ± 4	117 ± 9	79 ± 5	
Giada et al. [42]	Control (12)	58.0 ± 6.0	–	–	–	1.92 ± 0.1	69 ± 10	146 ± 17	83 ± 8	13
	Cyclists (12)	55.0 ± 5.0	–	–	–	1.86 ± 0.1	55 ± 6	139 ± 14	87 ± 8	
Kozakova et al. [44]	Control (16)	46.5 ± 16.0	–	–	–	–	82 ± 20	129 ± 12	67 ± 10	7
	Runners, triathletes (16)	53.1 ± 20.0	–	–	–	–	72 ± 8	135 ± 16	70 ± 12	
Lee et al. [45]	Control (9)	54.8 ± 4.3	182 ± 3	81.7 ± 5.0	24.8 ± 2.1	–	60 ± 4	114 ± 10	70 ± 10	13
	Cyclists, triathletes, speed-skaters (12)	53.8 ± 4.1	177 ± 7	75.1 ± 4.8	23.9 ± 2.1	–	57 ± 7	112 ± 10	69 ± 10	
Lindsay and Dunn [46]	Control (45)	52.0	–	–	–	1.83 ± 0.27	–	129 ± 17	77 ± 13	14
	Runners (45)	52.0 ± 11.4	–	–	–	1.88 ± 0.13	–	129 ± 16	77 ± 8	
Maufrais et al. [50]	Control (26)	56.0 ± 6.0	175 ± 6	80.9 ± 22.4	26.3 ± 0.2	1.95 ± 0.22	65 ± 10	128 ± 11	82 ± 7	14
	Runners, triathletes, cyclists (35)	54.0 ± 7.0	175 ± 14	73.7 ± 7.9	25.2 ± 8.6	1.88 ± 0.15	56 ± 9	127 ± 11	80 ± 9	
Northcote et al. [54]	Control (17)	56.0 ± 7.0	174 ± 8	74.0 ± 8.0	24.5 ± 3.5	–	72 ± 10	135 ± 23	–	12
	Runners (18)	56.0 ± 7.0	175 ± 5	64.0 ± 5.0	22.4 ± 0.1	–	51 ± 7	125 ± 15	–	
Nottin et al. [55]	Control (14)	55.9 ± 4.1	173 ± 4	80.4 ± 5.5	–	1.94 ± 0.08	73 ± 10	135 ± 9	83 ± 7	13
	Cyclists (14)	58.6 ± 4.8	173 ± 7	73.0 ± 9.4	–	1.86 ± 0.15	63 ± 6	141 ± 6	83 ± 8	
Sagiv et al. [57]	Control (15)	60.1 ± 1.1	173 ± 5	72.1 ± 3.0	–	–	81 ± 8	122 ± 6	82 ± 6	12
	Aerobic athletes (15)	59.3 ± 1.1	175 ± 7	71.4 ± 3.2	–	–	69 ± 9	117 ± 7	77 ± 10	
Nishimura et al. [53]	Control (15)	46.9 ± 3.3	–	–	–	1.62 ± 0.05	60 ± 8	127 ± 13	75 ± 9	14
	Bicyclists (29)	45.6 ± 2.3	–	–	–	1.78 ± 0.10	54 ± 7	120 ± 10	78 ± 8	
Baldi et al. [29]	Control (20)	65.7 ± 3.7	176 ± 4	77.5 ± 10.3	25.1 ± 2.6	1.96 ± 0.15	63 ± 9	140 ± 8	78 ± 17	11
	Endurance athletes (19)	65.2 ± 4.2	175 ± 6	70.1 ± 6.8	23.1 ± 1.8	1.82 ± 0.42	56 ± 6	126 ± 7	80 ± 6	
Bouvier et al. [31]	Control (12)	74.9 ± 2.4	175 ± 6	78.4 ± 11.0	25.8 ± 3.5	1.93 ± 0.13	69 ± 9	148 ± 14	81 ± 7	9
	Orienteers, runners (10)	72.8 ± 2.9	179 ± 3	72.5 ± 8.7	22.6 ± 2.1	1.90 ± 0.13	58 ± 11	151 ± 26	78 ± 7	
Carrick-Ranson et al. [32]	Control (27)	66.0 ± 5.0	176 ± 7	87.0 ± 16.0	28.0 ± 5.0	–	69 ± 11	–	–	11
	Cyclists, runners, dual/triathletes (17)	66.0 ± 4.0	176 ± 6	75.0 ± 10.0	24.0 ± 2.0	–	62 ± 10	–	–	
Di Bello et al. [36]	Control (11)	69.7 ± 8.4	169 ± 4	64.8 ± 9.5	–	1.80 ± 0.10	83 ± 21	145 ± 8	81 ± 3	13
	Runners (12)	65.7 ± 7.1	176 ± 8	69.2 ± 9.1	–	1.90 ± 0.20	68 ± 15	145 ± 6	81 ± 2	
Galetta et al. [40]	Control (16)	66.9 ± 4.6	177 ± 12	77.3 ± 8.7	24.6 ± 1.1	–	68 ± 7	134 ± 8	82 ± 4	15
	Runners (16)	67.6 ± 4.5	177 ± 15	74.8 ± 6.5	23.7 ± 0.7	–	54 ± 7	125 ± 6	79 ± 8	
Galetta et al. [39]	Control (25)	68.3 ± 3.2	176 ± 7	76.8 ± 5.6	24.5 ± 1.1	–	68 ± 6	133 ± 6	82 ± 4	15
	Runners (25)	69.4 ± 3.8	175 ± 7	75.9 ± 5.6	24.7 ± 0.7	–	54 ± 5	130 ± 5	81 ± 6	
Gates et al. [41]	Control (11)	65.0 ± 6.6	177 ± 7	85.0 ± 16.6	–	2.02 ± 0.17	59 ± 7	119 ± 17	74 ± 7	13
	Aerobic athletes (12)	68.0 ± 6.9	174 ± 3	74.0 ± 6.9	–	1.89 ± 0.17	48 ± 7	128 ± 17	76 ± 10	
Gates et al. [41]	Control (24)	51.0 ± 4.9	177 ± 5	85.0 ± 14.7	–	2.01 ± 0.15	60 ± 10	116 ± 10	73 ± 5	13
	Aerobic athletes (16)	50.0 ± 8.0	176 ± 8	73.0 ± 8.0	–	1.89 ± 0.16	47 ± 8	112 ± 8	68 ± 4	
Jungblut et al. [43]	Control (12)	69.0 ± 3.0	173 ± 9	79.0 ± 16.0	26 ± 3.4	–	69 ± 17	144 ± 17	82 ± 9	13
	Runners (12)	69.0 ± 5.0	173 ± 5	66.0 ± 6.2	22 ± 1.7	–	57 ± 8	141 ± 15	77 ± 6	
Schmidt et al. [58]	Control (26)	68.2 ± 3.2	176 ± 3	84 .1 ± 11.1	27.2 ± 3.8	–	61 ± 9	129 ± 14	77 ± 9	14
	Soccer players (17)	68.1 ± 2.1	178 ± 2	78.1 ± 8.2	24.6 ± 2.3	–	55 ± 8	127 ± 14	75 ± 9	
Seals et al. [59]	Control (6)	63.0 ± 3.0	181 ± 2	81.3 ± 7.4	–	2.01 ± 0.07	71 ± 7	130 ± 7	–	11
	Runners (8)	64.0 ± 6.0	173 ± 6	63.8 ± 5.7	–	1.76 ± 0.08	50 ± 6	137 ± 14	–	
Molmen et al. [52]	Control (10)	71.7 ± 1.3	–	76.5 ± 9.4	25 ± 2.5	–	66 ± 9	145 ± 17	81 ± 9	15
	Cross–country skiers (11)	74.3 ± 1.8	–	74.5 ± 8.3	23 ± 1.9	–	53 ± 8	122 ± 16	71 ± 9	
Cottini et al. [34]	Control (15)	61.0 ± 7.0	–	–	–	–	73 ± 8	128 ± 10	80 ± 8	6
	Aerobic athletes (15)	60.0 ± 10.0	–	–	–	–	58 ± 8	120 ± 9	75 ± 8	
Olsen et al. [56]	Control (11)	66.3 ± 3.8	175 ± 4	75.1 ± 4.4	24.6 ± 1.6	–	63 ± 7	127 ± 10	78 ± 5	15
	Runners (17)	65.0 ± 4.6	176 ± 4	71.1 ± 5.9	23.1 ± 1.6	–	54 ± 8	138 ± 18	76 ± 14	
Fleg et al. [38]	Control (23)	63.0 ± 6.0	177 ± 6	79.7 ± 11.9	25.3 ± 2.8	1.97 ± 0.16	72 ± 9	131 ± 17	79 ± 11	15
	Runners (16)	65.0 ± 8.0	176 ± 6	70.7 ± 8.1	22.8 ± 2.2	1.86 ± 0.12	56 ± 12	125 ± 17	74 ± 14	
Miki et al. [51]	Control (14)	49.0 ± 7.6	–	–	–	1.62 ± 0.14	61 ± 9	122 ± 10	–	10
	Cyclists (35)	49.4 ± 6.4	–	–	–	1.82 ± 0.10	51 ± 7	125 ± 9	–	
Matelot et al. [48]	Control (10)	59.0 ± 3.0	173 ± 5	78.6 ± 12.3	26.1 ± 3.2	–	70 ± 9	123 ± 8	79 ± 8	12
	Runners, cyclists (13)	62.0 ± 3.0	172 ± 4	71.2 ± 6.1	24.1 ± 2.1	–	57 ± 10	120 ± 12	78 ± 9	
Donal et al. [37]	Control (15)	58.9 ± 8.6	175 ± 10	78 ± 12.6	–	1.90 ± 0.20	–	141 ± 12	83 ± 7	14
	Cyclists (38)	61.5 ± 5.6	173 ± 10	72.3 ± 6.3	–	1.85 ± 0.10	–	137 ± 10	80 ± 8	
Bohm et al. [30]	Control (33)	46.0 ± 9.0	181 ± 6	80.0 ± 7.0	–	2.00 ± 0.10	65 ± 11	127 ± 8	78 ± 6 75 ± 7	15
	Runners, rowers, triathletes (33)	47.0 ± 8.0	182 ± 5	75.0 ± 6.0	–	1.96 ± 0.10	48 ± 7	128 ± 8		
Grace et al. [7]	Control (22)	62.7 ± 5.2	175 ± 6	89.9 ± 17.2	–	–	65 ± 12	139 ± 10	87 ± 5	13
	Triathletes, athletics, sprint cyclists, racquet sports (17)	61.1 ± 5.4	173 ± 5.5	79.5 ± 12.3	–	–	59 ± 10	131 ± 11	82 ± 7	
Maufrais et al. [49]	Control (20)	58.0 ± 7.0	177 ± 6	75.5 ± 11.8	–	1.92 ± 0.16	65 ± 9	127 ± 11	84 ± 7	13
	Cyclists (22)	61.0 ± 7.0	177 ± 7	73.3 ± 8	–	1.90 ± 0.12	53 ± 9	127 ± 11	84 ± 7	
Maessen et al. [47]	Control (13)	55.0 ± 8.0	181 ± 5	88.0 ± 9.0	–	2.09 ± 0.10	–	–	–	13
	Endurance athletes (16)	57.0 ± 8.0	178 ± 8	74.0 ± 8.0	–	1.91 ± 0.13	–	–	–	

Data are means ± standard deviation

*BMI* body mass index, *BSA* body surface area, *DBP* diastolic blood pressure, *HR* heart rate, *SBP* systolic blood pressure

**Table 2:** Descriptions of athlete training programmes and control activity levels as reported by studies

References	Controls		Athletes					
	Activity levels	Aerobic capacity (mL·min·kg)	Sport	Training frequency	Training volume	Training intensity	Training years	Aerobic capacity (mL·min·kg)
Child et al. [33]	Sedentary	35.0 ± 5.0	Runners	–	–	–	6 athletes ≥ 10	56.0 ± 8.0
D’Andrea et al. [35]	Sedentary	–	Swimmers	15–20 h/week. 3 h/day swimming and 3 h /week LDR	–	70–90% heart rate maximum	> 10	–
Giada et al. [42]	Not engaged in training program	23.0 ± 3.0	Cyclists	–	213 ± 89 km/week in previous 6 months	–	21 ± 12	43.0 ± 7.0
Kozakova et al. [44]	Untrained	–	Runners, triathletes	–	–	–	28 ± 15	–
Lee et al. [45]	< 2 h of aerobic exercise per week	33.8 ± 5.0	Cyclists, triathletes, speed-skaters	> 6 h/week for the last 3 years	–	≥ 50% of session performed at ≥ 60% $\dot{V}{\text{O}}_{{2{ \hbox{max} }}}$	≥ 3	47.9 ± 4.3
Lindsay and Dunn [46]	Sedentary	–	Runners	–	48 ± 25.76 km/week	–	20 ± 13	–
Maufrais et al. [50]	No regular training habits	–	Runners, triathletes, cyclists	> 8 h/week	–	–	24 ± 12	–
Northcote et al. [54]	Sedentary lifestyle. Participation in golf, walking and bowls	–	Runners	–	47 ± 23 miles/week	–	36 ± 7	–
Nottin et al. [55]	Sedentary	–	Cyclists	10.5 ± 2.3 h/week	11000 ± 3000 km/year	–	22 ± 5	–
Sagiv et al. [57]	Untrained	31.1 ± 2.4	Aerobic athletes	–	–	–	≥ 18 months	42.1 ± 2.1
Nishimura et al. [53]	Sedentary	–	Bicyclists	1–2 h/day, 4–5 days/week	–	–	27 ± 2	–
Baldi et al. [29]	No participation in endurance sports previous 2 years. Some participation in recreational sports	31.5 ± 5.9	Endurance athletes	–	–	–	21 ± 9	49.7 ± 6.8
Bouvier et al. [31]	Sedentary or moderately active subjects	26.0 ± 5.0	Orienteers, runners	3–7 h/week	–	Strenuous	15–25 years of age	41.0 ± 7.0
Carrick-Ranson et al. [32]	≤ 60 per week of endurance type training in last 12 months	27.0 ± 4.0	Cyclists, runners, dual/triathletes	4 × 45 min/week (at least 3 h/week) over the previous 12 months	–	–	≥ 1	45.0 ± 5.0
Di Bello et al. [36]	Sedentary lifestyles	–	Runners	1–2 h/day, 5 days/week	–	–	–	–
Galetta et al. [40]	Sedentary lifestyles with no regular exercise. $\dot{V}{\text{O}}_{{2{ \hbox{max} }}}$ < 45 mL·min·kg	38.0 ± 7.1	Runners	1–2 h of activity/day, 5 days/week. LDR 3 days and walking weight training 2 days	–	–	≥ 40	63.7 ± 3.4
Galetta et al. [39]	$\dot{V}{\text{O}}_{{2{ \hbox{max} }}}$ < 35 mL·min·kg	28.0 ± 4.1	Runners	> 5 times/week	–	Vigorous	37 ± 5	45.7 ± 3.4
Gates et al. [41]	No regular aerobic exercise	29.0 ± 10.0	Aerobic athletes	> 5 times/week	–	–	24 ± 4	37.0 ± 6.9
Gates et al. [41]	No regular aerobic exercise	33.0 ± 4.9	Aerobic athletes	> 5 times/week	–	–	19 ± 3	51.0 ± 8.0
Jungblut et al. [43]	No sporting participation in previous 6 months	27.0 ± 4.7	Runners	–	40 miles/week for at least previous 6 months	–	≥ 6 months	44.0 ± 6.3
Schmidt et al. [58]	Habitually active with no prior participation in structured exercise training	29.0 ± 4.9	Soccer players	1.5 ± 0.6 1-h session/week	26 ± 12 matches (2 × 35 min) per year	–	52 ± 11	34.1 ± 5.0
Seals et al. [59]	No regular training habits for several years	29.6 ± 3.43	Runners	–	43 ± 18 miles/week	–	10 ± 6	50.4 ± 4.8
Molmen et al. [52]	Exercise < 2 times per week in previous year	35.0 ± 5.0	Cross-country skiers	> 5 times/week	–	–	≥ 25	49.5 ± 4.5
Cottini et al. [34]	Sedentary	–	Aerobic athletes	–	–	–	–	–
Olsen et al. [56]	Sedentary for at least the last 5 years	29.6 ± 3.1	Runners	4–6 days/week	49 ± 12 km/week	Moderate to high	29 ± 8	46.9 ± 7.0
Fleg et al. [38]	No regular participation in endurance exercise. $\dot{V}{\text{O}}_{{2{ \hbox{max} }}}$ within 1 SD of age-adjusted sedentary values of BLISA men	30.2 ± 5.0	Runners	–	160 (median) km/month	–	16 (median)	47.2 ± 5.9
Miki et al. [51]	Untrained	–	Cyclists	–	–	–	29	–
Matelot et al. [48]	≤ 2 h of physical activity throughout life and never involved in competitive sport	33.0 ± 4.3	Runners, cyclists	7 ± 4 h/week	–	–	39 ± 4	47.3 ± 7.1
Donal et al. [37]	< 2 h of training per week	–	Cyclists	> 8 h/week	–	–	≥ 5	47.8 ± 5.7
Bohm et al. [30]	≤ 3 h/week	37.0 ± 6.0	Runners, rowers, triathletes	> 10 h/week	–	–	29 ± 8	60.0 ± 5.0
Grace et al. [7]	No participation in any formal exercise	–	Triathletes, athletics, sprint cyclists, racquet sports	–	–	–	Lifelong	–
Maufrais et al. [49]	No regular training habits	33.3 ± 7.2	Cyclists	9 ± 2 h/week	–	–	31 ± 11	42.3 ± 5.9
Maessen et al. [47]	< 10 MET-h/week over the last 20 years	31.6 ± 4.8	Endurance athletes	7.1 (median) h/week		60 (median) MET-h/week	≥ 20 (lifelong)	48.0 ± 8.9

Data are presented as mean ± SD unless otherwise stated

*BLISA* Baltimore Longitudinal Study of Aging, *LDR* long-distance running, *MET* metabolic equivalent of task, *SD* standard deviation, $\dot{V}O_{2max}$ maximal oxygen uptake

**Table 3:** Summary of athlete and control groups included for measures of cardiac structure

References	Study group (*n*)	IVS (mm)	PWT (mm)	LVEDD (mm)	LVM (g)	LVMi (g·m^2^)	RWT	LVEDV (mL)	LAD (mm)	RVEDD (mm)
Child et al. [33]	Control (12) Runners (9)	10.5 ± 1.5 11.2 ± 2.2^↔^	9.9 ± 0.8 10.3 ± 0.9^↔^	48.3 ± 4.5 52.1 ± 3.7^↑^	– –	111.7 ± 23.8 153.9 ± 27.4^↑^	– –	– –	– –	– –
D’Andrea et al. [35]	Control (25) Swimmers (40)	9.3 ± 1.1 10.2 ± 2.1^↑^	8.4 ± 2.1 9.4 ± 2.1^↔^	47.4 ± 4.7 56.4 ± 4.7^↑^	– –	– –	0.43 ± 0.04 0.39 ± 0.04^↓^	– –	– –	21.2 ± 2.8 25.2 ± 3.8^↑^
Giada et al. [42]	Control (12) Cyclists (12)	– –	– –	– –	– –	93.0 ± 12.0 134.0 ± 19.0^↑^	– –	– –	– –	–
Kozakova et al. [44]	Control (16) Runners, triathletes (16)	9.8 ± 0.0 13.6 ± 0.0 (^a^)	9.6 ± 0.0 12.5 ± 0.0 (^a^)	49.0 ± 8.0 52.8 ± 4.0^↔^	191.4 ± 56.0 348.6 ± 69.2^↑^	94.9 ± 22.0 184.6 ± 33.6^↑^	– –	– –	– –	–
Lee et al. [45]	Control (9) Cyclists, triathletes, speed-skaters (12)	9.0 ± 2.0 9.0 ± 3.0^↔^	10.0 ± 6.0 10.0 ± 4.0^↔^	45.0 ± 10.0 45.0 ± 8.0^↔^	– –	58.4 ± 8.0 90.3 ± 15.0^↑^	0.36 ± 0.05 0.38 ± 0.01^↔^	108.0 ± 22.0 97.0 ± 29.0^↔^	– –	–
Lindsay and Dunn [46]	Control (45) Runners (45)	9.0 ± 2.0 12.0 ± 2.0^↑^	9.0 ± 1.3 11.0 ± 1.3^↑^	48.0 ± 4.7 52.0 ± 4.0^↑^	– –	97.0 ± 20.1 141.0 ± 33.5^↑^	0.37 ± 0.07 0.42 ± 0.07^↑^	– –	– –	–
Maufrais et al. [50]	Control (26) Runners, triathletes, cyclists (35)	– –	– –	50.0 ± 5.0 55.0 ± 4.0^↑^	– –	– –	– –	– –	– –	–
Northcote et al. [54]	Control (17) Runners (18)	11.0 ± 0.8 12.2 ± 2.2^↑^	10.8 ± 0.9 12.1 ± 1.9^↑^	46.9 ± 4.1 46.8 ± 6.6^↔^	221.0 ± 36.7 265.0 ± 84.2^↑^	118.0 ± 17.6 151.0 ± 50.4^↑^	0.46 ± 0.06 0.53 ± 0.11^↑^	104.0 ± 18.4 112.0 ± 24.1^↔^	– –	–
Nottin et al. [55]	Control (14) Cyclists (14)	– –	– –	– –	– –	98.0 ± 22.0 104.0 ± 23.0^↔^	– –	– –	– –	–
Nishimura et al. [53]	Control (15) Bicyclists (29)	8.6 ± 1.1 11.0 ± 1.5^↑^	8.4 ± 1.0 10.1 ± 1.4^↑^	48.6 ± 2.4 54.1 ± 3.0^↑^	147.0 ± 23.0 222.0 ± 35.0^↑^	– –	– –	132.0 ± 15.0 173.0 ± 24.0^↑^	40.0 ± 5.6 31.1 ± 5.4^↑^	–
Baldi et al. [29]	Control (20) Endurance athletes (19)	7.1 ± 0.4 8.6 ± 0.3^↑^	7.3 ± 0.3 8.9 ± 0.3^↑^	49.6 ± 4.2 54.1 ± 3.1^↑^	155.0 ± 6.4 174.7 ± 30.6^↑^	78.4 ± 24.3 94.3 ± 17.5^↑^	– –	– –	34.7 ± 8.6 35.2 ± 4.4^↔^	–
Bouvier et al. [31]	Control (12) Orienteers, runners (10)	11.0 ± 1.3 11.0 ± 1.5^↔^	11.0 ± 1.4 12.0 ± 0.8^↑^	46.0 ± 6.0 48.0 ± 4.0^↔^	– –	121.0 ± 27.0 136.0 ± 24.0^↔^	– –	– –	39.0 ± 4.0 39.0 ± 4.0^↔^	–
Carrick-Ranson et al. [32]	Control (27) Cyclists, runners, dual/triathletes (17)	– –	– –	55.0 ± 5.0 55.0 ± 6.0^↔^	166.0 ± 40.0 203.0 ± 41.0^↑^	– –	– –	– –	– –	–
Di Bello et al. [36]	Control (11) Runners (12)	10.9 ± 2.3 13.1 ± 1.9^↑^	10.6 ± 1.4 12.5 ± 1.9^↑^	48.1 ± 5.2 50.7 ± 5.1^↔^	225.2 ± 62.7 318.9 ± 81.2^↑^	123.2 ± 29.1 165.1 ± 36.7^↑^	– –	– –	34.0 ± 3.2 35.9 ± 4.8^↔^	–
Galetta et al. [40]	Control (16) Runners (16)	10.1 ± 0.7 12.2 ± 1.4^↑^	9.6 ± 0.8 11.9 ± 1.2^↑^	49.1 ± 4.6 53.2 ± 4.2^↑^	148.2 ± 12.5 258.2 ± 14.2^↑^	– –	– –	– –	– –	– –
Galetta et al. [39]	Control (25) Runners (25)	10.1 ± 0.6 12.2 ± 1.3^↑^	9.4 ± 0.7 11.9 ± 1.1^↑^	49.7 ± 4.4 53.8 ± 4.4^↑^	146.2 ± 11.5 256.2 ± 12.8^↑^	– –	– –	– –	– –	– –
Gates et al. [41]	Control (11) Aerobic athletes (12)	– –	– –	– –	– –	85.0 ± 16.6 106.0 ± 17.3^↑^	0.40 ± 0.04 0.38 ± 0.04^↔^	– –	– –	– –
Gates et al. [41]	Control (24) Aerobic athletes (16)	– –	– –	– –	– –	85.0 ± 9.8 98.0 ± 12.0^↑^	0.37 ± 0.04 0.35 ± 0.02^↔^	– –	– –	– –
Jungblut et al. [43]	Control (12) Runners (12)	9.0 ± 1.0 9.0 ± 2.0^↔^	10.0 ± 2.0 10.0 ± 1.0 (↔	47.0 ± 5.0 53.0 ± 6.0^↑^	– –	51.0 ± 7.8 61.0 ± 13.0^↑^	0.40 ± 0.08 0.40 ± 0.07^↔^	– –	– –	– –
Schmidt et al. [58]	Control (26) Soccer players (17)	10.0 ± 2.0 10.0 ± 1.0^↔^	10.0 ± 2.0 9.0 ± 1.0^↔^	49.0 ± 5.0 50.0 ± 5.0^↔^	– –	85.4 ± 17.3 90.6 ± 21.2^↔^	– –	97.0 ± 20.0 116.0 ± 22.0^↑^	– –	– –
Seals et al. [59]	Control (6) Runners (8)	– –	– –	– –	– –	– –	0.35 ± 0.02 0.34 ± 0.06^↔^	– –	– –	– –
Cottini et al. [34]	Control (15) Aerobic athletes (15)	9.1 ± 1.0 9.4 ± 0.8^↔^	8.5 ± 1.1 8.8 ± 0.7^↔^	52.8 ± 3.1 54.0 ± 2.0^↔^	– –	– –	– –	138.0 ± 8.4 146.0 ± 8.4^↑^	36.0 ± 3.9 35.0 ± 4.8^↔^	– –
Olsen et al. [56]	Control (11) Runners (17)	– –	– –	45.0 ± 2.0 50.0 ± 4.0^↑^	129.0 ± 20.0 188.0 ± 39.0^↑^	68.0 ± 11.0 101.0 ± 20.0^↑^	0.38 ± 0.04 0.37 ± 0.06^↔^	89.0 ± 18.0 122.0 ± 15.0^↑^	– –	– –
Fleg et al. [38]	Control (23) Runners (16)	– –	– –	– –	– –	102.0 ± 30.0 105.0 ± 30.0^↔^	0.48 ± 0.10 0.42 ± 0.10^↔^	– –	– –	– –
Miki et al. [51]	Control (14) Cyclists (35)	– –	– –	– –	– –	107.5 ± 17.4 180.9 ± 34.2^↑^	0.39 ± 0.04 0.47 ± 0.09^↑^	– –	– –	– –
Matelot et al. [48]	Control (10) Runners, cyclists (13)	9.4 ± 1.4 9.9 ± 1.6^↔^	7.9 ± 1.5 9.6 ± 1.8^↑^	48.6 ± 2.8 52.5 ± 3.7^↑^	– –	– –	– –	– –	– –	– –
Donal et al. [37]	Control (15) Cyclists (38)	9.0 ± 1.0 11.0 ± 1.0^↑^	10.0 ± 2.0 11.0 ± 1.0^↑^	47.0 ± 4.0 48.0 ± 4.0^↔^	– –	79.3 ± 15.7 104.7 ± 17.2^↑^	– –	117.5 ± 23.2 120.5 ± 20.6^↔^	– –	20.5 ± 3.7 20.1 ± 2.7^↔^
Bohm et al. [30]	Control (33) Runners, rowers, triathletes (33)	10.3 ± 0.8 11.7 ± 0.7^↑^	9.2 ± 1.1 10.4 ± 1.1^↑^	50.0 ± 4.2 56.4 ± 2.2^↑^	– –	– –	– –	– –	– –	27.3 ± 4.3 34.1 ± 3.8^↑^
Sagiv et al. [57]	Control (15) Aerobic athletes (15)	– –	– –	– –	– –	– –	– –	100.7 ± 6.2 108.1 ± 5.8^↑^	– –	– –
Molmen et al. [52]	Control (10 Cross-country skiers (11)	– –	– –	– –	– –	– –	– –	102.0 ± 13.0 142.0 ± 21.0^↑^	– –	– –
Grace et al. [7]	Control (22) Triathletes, athletics, sprint cyclists, racquet sports (17)	10.0 ± 1.5 10.0 ± 1.0^↔^	9.7 ± 1.3 10.5 ± 1.3^↔^	51.4 ± 5.2 52.7 ± 4.8^↔^	223.0 ± 48.0 249.0 ± 49.0^↔^	89.0 ± 16.9 96.0 ± 16.2^↔^	– –	– –	– –	– –
Maessen et al. [47]	Control (13) Endurance athletes (16)	– –	– –	– –	– –	– –	– –	92.0 ± 15.0 101.0 ± 24.0	– –	– –
Maufrais et al. [49]	Control (20) Cyclists (22)	– –	– –	51.0 ± 6.0 55.0 ± 5.0^↑^	– –	96.0 ± 26.0 127.0 ± 27.0^↑^	– –	– –	– –	– –

Data are mean ± standard deviation unless otherwise stated

Statistical significance at *p* < 0.05

*IVS* interventricular septal, *LAD* left atrial diameter, *LVEDD* left ventricular end-diastolic diameter, *LVEDV* left ventricular end-diastolic volume, *LVM* left ventricular mass, *LVMi* left ventricular mass index, *n* participant number, *PWT* posterior wall thickness, *RVEDD* right ventricular end-diastolic diameter, *RWT* relative wall thickness

^↑^Significantly greater in athletes

^↓^Significantly less in athletes

^↔^No athlete–control difference

^a^Not included in meta-analysis

**Table 4:** Summary of athlete and control groups included for measures of left ventricular systolic function

References	Study group (*n*)	EF (%)	FS (%)	SV (mL)	*s*′ (cm·s^−1^)	GLS (%)
Child et al. [33]	Control (12) Runners (9)	– –	39.1 ± 4.7 37.8 ± 2.6^↔^	– –	– –	– –
D’Andrea et al. [35]	Control (25) Swimmers (40)	– –	41.7 ± 3.7 46.7 ± 4.7^↑^	71.4 ± 3.2 90.1 ± 6.2^↑^	9.0 ± 4.0 14.0 ± 3.0^↑^	– –
Giada et al. [42]	Control (12) Cyclists (12)	67.0 ± 4.0 63.0 ± 5.0^↓^	– –	– –	– –	– –
Lee et al. [45]	Control (9) Cyclists, triathletes, speed-skaters (12)	61.2 ± 2.4 61.6 ± 4.0^↔^	– –	– –	– –	– –
Maufrais et al. [50]	Control (26) Runners, triathletes, cyclists (35)	– –	– –	– –	7.3 ± 1.6 7.5 ± 1.6^↔^	– –
Northcote et al. [54]	Control (17) Runners (18)	58.0 ± 7.0 57.0 ± 10.5^↔^	27.0 ± 6.2 29.0 ± 5.7^↔^	– –	– –	– –
Nottin et al. [55]	Control (14) Cyclists (14)	61.0 ± 3.0 62.0 ± 2.0^↔^	– –	– –	10.5 ± 2.4 9.6 ± 1.8^↔^	– –
Sagiv et al. [57]	Control (15) Aerobic athletes (15)	– –	– –	57.3 ± 6.6 68.1 ± 4.3^↑^	– –	– –
Nishimura et al. [53]	Control (15) Bicyclists (29)	66.0 ± 2.0 63.0 ± 4.0^↓^	32.0 ± 2.0 30.0 ± 3.0^↓^	93.0 ± 16.0 111.0 ± 15.0^↑^	– –	– –
Baldi et al. [29]	Control (20) Endurance athletes (19)	– –	36.9 ± 5.7 33.7 ± 5.6^↔^	– –	7.1 ± 0.9 8.3 ± 1.4^↑^	– –
Bouvier et al. [31]	Control (12) Orienteers, runners (10)	– –	32.0 ± 6.0 31.0 ± 6.0^↔^	– –	– –	– –
Carrick-Ranson et al. [32]	Control (27) Cyclists, runners, dual/triathletes (17)	– –	– –	– –	10.0 ± 1.0 8.0 ± 2.0^↓^	– –
Di Bello et al. [36]	Control (11) Runners (12)	71.8 ± 9.1 75.5 ± 9.3^↔^	42.0 ± 8.2 45.2 ± 9.0^↔^	65.5 ± 36.1 97.3 ± 23.2^↑^	– –	– –
Galetta et al. [40]	Control (16) Runners (16)	67.2 ± 4.5 64.2 ± 5.2^↔^	– –	– –	– –	– –
Galetta et al. [39]	Control (25) Runners (25)	66.2 ± 4.5 64.2 ± 5.2^↔^	– –	– –	8.9 ± 0.8 9.3 ± 0.8^↔^	– –
Jungblut et al. [43]	Control (12) Runners (12)	– –	40.0 ± 6.0 40.0 ± 4.0^↔^	– –	– –	– –
Schmidt et al. [58]	Control (26) Soccer players (17)	54.0 ± 6.0 58.0 ± 4.0^↑^	– –	– –	8.7 ± 1.5 8.1 ± 1.3^↔^	**− **17.7 ± 2.5 **− **19.9 ± 2.5^↑^
Seals et al. [59]	Control (6) Runners (8)	– –	36.2 ± 6.9 33.3 ± 8.8^↔^	– –	– –	– –
Molmen et al. [52]	Control (10) Cross-country skiers (11)	58.7 ± 7.2 63.7 ± 4.8^↔^	– –	79.0 ± 13.0 102.0 ± 25.0^↑^	7.3 ± 0.8 8.2 ± 1.6^↔^	– –
Cottini et al. [34]	Control (15) Aerobic athletes (15)	58.0 ± 2.7 65.0 ± 3.0^↑^	– –	– –	– –	– –
Olsen et al. [56]	Control (11) Runners (17)	59.0 ± 3.0 60.0 ± 4.0^↔^	– –	– –	9.4 ± 1.2 8.2 ± 1.5^↓^	– –
Fleg et al. [38]	Control (23) Runners (16)	– –	42.0 ± 9.0 38.0 ± 6.0^↔^	– –	– –	– –
Miki et al. [51]	Control (14) Cyclists (35)	– –	35.6 ± 3.7 34.7 ± 4.2^↔^	– –	– –	– –
Matelot et al. [48]	Control (10) Runners, cyclists (13)	67.1 ± 5.6 62.2 ± 4.3^↓^	– –	– –	8.9 ± 1.5 8.6 ± 2.0^↔^	**− **19.4 ± 2.1 **− **19.8 ± 1.9^↔^
Donal et al. [37]	Control (15) Cyclists (38)	62.8 ± 6.8 61.4 ± 6.0^↔^	– –	76.2 ± 14.3 74.0 ± 14.5^↔^	10.2 ± 1.3 9.2 ± 2.2^↔^	**− **18.0 ± 2.4 **− **17.3 ± 2.2^↔^
Bohm et al. [30]	Control (33/32^a^) Runners, rowers, triathletes (33/32 ^a^)	– –	– –	– –	9.0 ± 1.5 9.5 ± 1.5^↔^	**− **18.0 ± 2.0^a^ **− **17.0 ± 2.0^a↓^
Grace et al. [7]	Control (22) Triathletes, athletics, sprint cyclists, racquet sports (17)	55.6 ± 8.6 60.9 ± 5.1^↑^	– –	67.0 ± 23.0 77.0 ± 9.0^↔^	– –	– –
Maessen et al. [47]	Control (13) Endurance athletes (18)	– –	– –	– –	9.0 ± 1.4 9.3 ± 1.9^↔^	– –
Maufrais et al. [49]	Control (20) Cyclists (22)	– –	– –	– –	7.8 ± 1.4 7.4 ± 1.2^↔^	18.1 ± 3.0 17.3 ± 2.1^↔^

Data are mean ± standard deviation unless otherwise stated

Statistical significance at *p* < 0.05

*EF* ejection fraction, *FS* fractional shortening, *GLS* global longitudinal strain, *n* participant number, *s*′ systolic tissue velocity, *SV* stroke volume

^↑^Significantly greater in athletes

^↓^Significantly less in athletes

^↔^No athlete–control different

^a^Different *n* for GLS

**Table 5:** Summary of athlete and control groups included for measures of left ventricular diastolic function

References	Study group (*n*)	*E* (cm·s^−1^)	*A* (cm·s^−1^)	*E*/*A*	*e*′ (cm·s^−1^)	*a*′ (cm·s^−1^)	*e*′/*a*′	*E*/*e*′
D’Andrea et al. [35]	Control (25) Swimmers (40)	72.0 ± 17.0 88.0 ± 12.0^↑^	53.0 ± 14.0 56.0 ± 12.0^↔^	1.4 ± 0.5 1.7 ± 0.5^↑^	9.0 ± 2.0 16.0 ± 4.0^↑^	11.0 ± 2.0 12.0 ± 2.0^↑^	0.8 ± 0.4 1.3 ± 0.3^↑^	– –
Giada et al. [42]	Control (12) Cyclists (12)	54.9 ± 12.0 57.0 ± 19.0^↔^	57.0 ± 7.0 54.0 ± 15.0^↔^	0.9 ± 0.2 1.0 ± 0.3^↔^	– –	– –	– –	– –
Lee et al. [45]	Control (9) Cyclists, triathletes, speed-skaters (12)	73.0 ± 22.0 73.0 ± 16.0^↔^	81.0 ± 13.0 62.0 ± 14.0^↓^	0.9 ± 0.2 1.2 ± 0.5^↔^	– –	– –	– –	– –
Lindsay and Dunn [46]	Control (45) Runners (45)	70.0 ± 20.0 70.0 ± 20.0^↔^	70.0 ± 20.0 60.0 ± 13.0^↓^	1.1 ± 0.2 1.2 ± 0.5(↔)	– –	– –	– –	– –
Maufrais et al. [50]	Control (26) Runners, triathletes, cyclists (35)	70.0 ± 15.0 71.0 ± 15.0^↔^	66.0 ± 13.0 64.0 ± 16.0^↔^	1.1 ± 0.2 1.2 ± 0.3^↔^	7.9 ± 1.3 8.8 ± 1.9^↑^	8.4 ± 1.3 7.5 ± 1.3^↓^	1.0 ± 0.2 1.2 ± 0.3^↑^	– –
Nottin et al. [55]	Control (14) Cyclists (!4)	53.7 ± 9.7 68.3 ± 13.1^↑^	67.4 ± 12.3 66.9 ± 10.6^↔^	0.8 ± 0.2 1.0 ± 0.2^↑^	11.3 ± 3.2 11.4 ± 2.0^↔^	12.4 ± 2.4 11.0 ± 1.7^↔^	0.9 ± 0.3 1.1 ± 0.3^↔^	4.4 ± 1.1 6.1 ± 2.0^↑^
Baldi et al. [29]	Control (20) Endurance athletes (19)	52.0 ± 10.9 56.6 ± 13.7^↔^	56.3 ± 11.2 57.2 ± 13.8^↔^	0.9 ± 0.3 1.0 ± 0.3^↔^	7.1 ± 1.5 7.7 ± 1.6^↔^	10.9 ± 1.5 11.9 ± 1.6^↑^	0.7 ± 0.1 0.7 ± 0.1^↔^	7.6 ± 2.0 7.5 ± 2.1^↔^
Bouvier et al. [31]	Control (12) Orienteers, runners (10)	– –	– –	0.8 ± 0.2 1.4 ± 0.7^↑^	– –	– –	– –	– –
Carrick-Ranson et al. [32]	Control (27) Cyclists, runners, dual/triathletes (17)	53.7 ± 15.3 53.7 ± 8.7^↔^	60.4 ± 20.7 48.8 ± 8.5^↓^	0.9 ± 0.2 1.1 ± 0.2^↑^	6.9 ± 1.4 6.6 ± 1.5^↔^	10.0 ± 2.1 8.5 ± 1.7^↓^	0.7 ± 0.1 0.8 ± 0.2^↔^	8.0 ± 2.0 9.0 ± 2.0^↔^
Di Bello et al. [36]	Control (11) Runners (12)	77.0 ± 10.0 84.0 ± 13.0^↔^	82.0 ± 22.0 74.0 ± 20.0^↔^	1.0 ± 0.2 1.2 ± 0.3^↑^	– –	– –	– –	– –
Galetta et al. [40]	Control (16) Runners (16)	56.6 ± 6.3 56.4 ± 6.1^↔^	59.8 ± 9.1 55.9 ± 8.5^↔^	0.9 ± 0.2 1.0 ± 0.3^↔^	– –	– –	– –	– –
Galetta et al. [39]	Control (25) Runners (25)	56.6 ± 6.3 58.4 ± 6.1^↔^	64.8 ± 8.1 45.9 ± 7.5^↓^	0.9 ± 0.2 1.2 ± 0.3^↑^	9.1 ± 2.3 12.3 ± 2.8^↑^	10.7 ± 1.7 8.6 ± 1.6^↓^	0.8 ± 0.3 1.4 ± 0.3^↑^	– –
Gates et al. [41]	Control (11) Aerobic athletes (12)	– –	– –	0.9 ± 0.2 1.1 ± 0.3^↔^	– –	– –	– –	– –
Gates et al. [41]	Control (24) Aerobic athletes (16)	– –	– –	1.3 ± 0.4 1.8 ± 0.5^↑^	– –	– –	– –	– –
Jungblut et al. [43]	Control (12) Runners (12)	76.0 ± 22.0 79.0 ± 16.0^↔^	75.0 ± 15.0 73.0 ± 19.0^↔^	1.0 ± 0.3 1.1 ± 0.2^↔^	– –	– –	– –	– –
Schmidt et al. [58]	Control (26) Soccer players (17)	70.0 ± 10.0 60.0 ± 10.0^↓^	70.0 ± 20.0 50.0 ± 10.0^↓^	1.1 ± 0.3 1.1 ± 0.2^↔^	10.2 ± 2.3 10.0 ± 1.9^↔^	11.8 ± 1.9 10.6 ± 2.4^↔^	– –	– –
Molmen et al. [52]	Control (10) Cross-country skiers (11)	58.0 ± 15.0 58.0 ± 14.0^↔^	71.0 ± 19.0 49.0 ± 14.0^↓^	0.9 ± 0.3 1.3 ± 0.7^↔^	6.9 ± 1.5 9.0 ± 2.1^↑^	11.5 ± 2.1 10.3 ± 2.8^↔^	– –	– –
Cottini et al. [34]	Control (15) Aerobic athletes (15)	62.2 ± 8.2 80.0 ± 13.6^↑^	68.2 ± 5.5 64.9 ± 12.3^↔^	0.9 ± 0.1 1.2 ± 0.1^↑^	– –	– –	– –	– –
Olsen et al. [56]	Control (11) Runners (17)	56.0 ± 8.0 63.0 ± 15.0^↔^	63.0 ± 12.0 59.0 ± 15.0^↔^	0.9 ± 0.2 1.1 ± 0.3^↔^	7.8 ± 1.7 8.3 ± 2.5^↔^	11.2 ± 1.0 10.1 ± 1.4^↓^	– –	7.5 ± 1.4 8.1 ± 2.8^↔^
Fleg et al. [38]	Control (23) Runners (16)	68.0 ± 19.0 56.0 ± 15.0^↓^	68.0 ± 19.0 51.0 ± 16.0^↓^	1.1 ± 0.4 1.2 ± 0.5^↔^	– –	– –	– –	– –
Matelot et al. [48]	Control (10) Runners, cyclists (13)	69.2 ± 8.3 64.0 ± 11.0^↔^	74.6 ± 16.5 58.9 ± 16.7^↓^	1.0 ± 0.2 1.1 ± 0.3^↔^	9.9 ± 1.9 10.5 ± 2.6^↔^	10.8 ± 1.9 10.5 ± 1.1^↔^	– –	7.2 ± 1.4 6.3 ± 1.3^↔^
Donal et al. [37]	Control (15) Cyclists (38)	71.5 ± 17.0 61.5 ± 13.3^↓^	68.0 ± 19.4 59.7 ± 13.8^↔^	1.1 ± 0.5 1.1 ± 0.3^↔^	12.3 ± 2.3 10.5 ± 2.3^↓^	11.2 ± 2.6 10.9 ± 2.6^↔^	– –	6.2 ± 1.7 6.2 ± 2.1^↔^
Bohm et al. [30]	Control (33) Runners, rowers, triathletes (33)	– –	– –	– –	12.0 ± 3.0 11.5 ± 3.0^↔^	10.5 ± 2.5 9.0 ± 2.5^↓^	1.25 ± 0.6 1.35 ± 0.6^↔^	6.0 ± 1.5 6.0 ± 1.5^↔^
Grace et al. [7]	Control (22) Triathletes, athletics, sprint cyclists, racquet sports (17)	68.0 ± 14.0 70.0 ± 11.0^↔^	63.0 ± 15.0 54.0 ± 7.0^↓^	1.1 ± 0.3 1.3 ± 0.3^↑^	6.2 ± 1.3 7.4 ± 1.4^↑^	– –	– –	– –
Maessen et al. [47]	Control (13) Endurance athletes (18)	62.7 ± 15.7 63.0 ± 11.9^↔^	– –	– –	– –	11.2 ± 2.0 11.3 ± 2.5^↔^	– –	– –
Maufrais et al. [49]	Control (20) Cyclists (22)	69.7 ± 11.3 73.0 ± 15.7^↔^	– –	– –	9.1 ± 1.6 9.5 ± 2.6^↔^	– –	– –	7.8 ± 1.6 7.8 ± 2.0^↔^

Data are presented as mean ± standard deviation unless otherwise stated

Statistical significance at *p* < 0.05

*A* late diastolic mitral inflow velocity, *a*′ late diastolic tissue velocity, *E* early diastolic mitral inflow velocity, *e*′ early diastolic tissue velocity, *e*′*/a*′ ratio of early to late diastolic tissue velocity, *E/A* ratio of early to late mitral inflow velocity, *E/e*′ ratio of early diastolic mitral inflow velocity to early diastolic tissue velocity, *n* participant number

^↑^Significantly greater in athletes

^↓^Significantly less in athletes

^↔^No athlete–control difference

**Table 6:** Meta-analyses of athlete–control differences for cardiac structure, systolic and diastolic function

Parameter	Number of studies	Difference in means	95% CI	*p* value	Heterogeneity		*p* value
					Cochran’s *Q*	*I*^2^ statistic (%)	
Cardiac structure							
IVS (mm)	18	1.23	0.85 to 1.60	**<** **0.01**	78.42	78.32	**<** **0.01**
PWT (mm)	18	1.20	0.83 to 1.56	**<** **0.01**	81.60	79.17	**<** **0.01**
LVEDD (mm)	23	3.65	2.66 to 4.64	**<** **0.01**	64.99	66.15	**<** **0.01**
RWT	11	0.00	−0.02 to 0.03	0.73	51.89	80.73	**<** **0.01**
LVM (g)	10	72.03	45.70 to 98.36	**<** **0.01**	211.29	95.74	**<** **0.01**
LVMi (g·m^2^)	20	28.17	19.84 to 36.49	**<** **0.01**	148.62	87.22	**<** **0.01**
LVEDV (mL)	10	16.11	7.80 to 24.43	**<** **0.01**	56.02	83.93	**<** **0.01**
LAD (mm)	5	2.07	−1.66 to 5.79	0.28	20.73	80.70	**<** **0.01**
RVEDD (mm)	3	3.49	−0.55 to 7.53	0.09	29.37	93.19	**<** **0.01**
Left ventricular systolic function							
EF (%)	15	0.43	−1.57 to 2.44	0.67	82.90	83.11	**<** **0.01**
FS (%)	11	−0.34	−2.32 to 1.63	0.73	34.60	71.10	**<** **0.01**
SV (mL)	7	13.59	7.20 to 19.98	**<** **0.01**	30.48	80.31	**<** **0.01**
*s*′ (cm·s^−1^)	14	0.09	−0.53 to 0.70	0.79	82.85	84.31	**<** **0.01**
GLS (%)	5	−0.04	−1.18 to 1.10	0.94	13.63	70.66	**0.01**
Left ventricular diastolic function							
*E* (cm·s^−1^)	22	2.08	−1.12 to 5.28	0.20	74.06	71.64	**<** **0.01**
*A* (cm·s^−1^)	20	−8.20	−11.90 to −4.51	**<** **0.01**	68.73	72.36	**<** **0.01**
*E*/*A*	23	0.18	0.13 to 0.24	**<** **0.01**	42.31	48.00	**0.01**
*e*′ (cm·s^−1^)	14	0.96	0.05 to 1.86	**0.04**	93.55	86.10	**<** **0.01**
*a*′ (cm·s^−1^)	13	−0.72	−1.31 to −0.12	**0.02**	41.32	70.96	**<** **0.01**
*e*′/*a*′	7	0.23	0.06 to 0.40	**0.01**	61.44	90.23	**<** **0.01**
*E*/*e*′	8	0.23	−0.31 to 0.77	0.40	12.51	44.06	0.08

Bold values indicate statistical significance (*p* < 0.05)

*A* late diastolic mitral inflow velocity, *a*′ late diastolic tissue velocity, *CI* confidence interval, *E* early diastolic mitral inflow velocity, *e*′ early diastolic tissue velocity, *e*′*/a*′ ratio of early to late diastolic tissue velocity, *E/A* ratio of early to late mitral inflow velocity, *E/e*′ ratio of early diastolic mitral inflow velocity to early diastolic tissue velocity, *EF* ejection fraction, *FS* fractional shortening, *GLS* global longitudinal strain, *IVS* interventricular septal, *LAD* left atrial diameter, *LVEDD* left ventricular end-diastolic diameter, *LVEDV* left ventricular end-diastolic volume, *LVM* left ventricular mass, *LVMi* left ventricular mass index, *PWT* posterior wall thickness, *RVEDD* right ventricular end-diastolic diameter, *RWT* relative wall thickness, *s*′ systolic tissue velocity, *SV* stroke volume

### Cardiac Structure

IVS, PWT, LVEDD, LVEDV, LVM and LVMi were greater in athletes compared with controls, while RWT, RVEDD and LAD did not differ. Significant heterogeneity was observed for all parameters, with inconsistency considered moderate for LVEDD, yet high for IVS, PWT, RWT, LVM, LVMi, LVEDV RVEDD and LAD.

### Left Ventricular Systolic Function

EF, FS, *s*′ and GLS were not different between athletes and controls, whereas SV was greater in athletes. Between-study heterogeneity was significant in all cases and the inconsistency considered moderate in FS and GLS, yet high for EF, *s*′ and SV.

### Left Ventricular Diastolic Function

Pooled analysis of studies measuring mitral inflow velocity revealed no difference in *E* between athletes and controls, albeit *A* was significantly lower in athletes. Accordingly, *E*/*A* was greater in athletes compared with controls. Tissue velocity analyses showed higher *e*′ in athletes, whereas athletes showed significantly reduced *a*′ but greater *e*′/*a*′ compared with controls. *E*/*e*′ was not different between athletes and controls. Between-study heterogeneity was significant for all parameters of diastolic parameters besides *E*/*e*′. Inconsistency was low for *E*/*A* and *E*/*e*′, moderate for *E*, *A* and *a*′, and high for *e*′ and *e*′/*a*′.

### Meta-Regression(s)

The difference between athletes and controls was maintained with chronological age for all available variables, with the exception of LVEDD, LVMi and *A* (Table 7). There was a significant inverse but opposite relationship in LVEDD, LVMi and *A* between athletes and controls that continued with chronological age.

**Table 7:** Meta-regression(s) of left ventricular structure and function between older athletes and controls during advancing age with interpretation

Covariate parameter	Number of studies	Cochran’s *Q*	SE	*β*	95% CI	*p* value	Interpretation (i.e. difference between athletes and controls with advancing age)
Left ventricular structure							
IVS	18	1.88	0.02	−0.04	−0.08 to 0.01	0.17	Maintained
PWT	18	0.05	0.02	−0.005	−0.05 to 0.04	0.82	Maintained
LVEDD	23	6.42	0.05	−0.14	−0.24 to −0.03	**0.01**	Reduced. The greater LVEDD in athletes reduced by ~ 0.14 mm per year relative to controls
RWT	11	1.06	0.002	−0.002	−0.01 to 0.002	0.30	Maintained
LVM	10	0.29	1.81	−0.97	−4.51 to 2.58	0.59	Maintained
LVMi	20	9.79	0.57	−1.79	−2.91 to −0.67	**<** **0.01**	Reduced. The greater LVMi in athletes reduced by ~ 1.79 g·m^2^ per year relative to controls
LVEDV	10	0.44	0.62	0.41	−0.81 to 1.63	0.51	Maintained
Left ventricular systolic function							
EF	15	1.57	0.15	0.19	−0.11 to 0.48	0.21	Maintained
FS	11	0.42	0.11	−0.07	−0.30 to 0.15	0.51	Maintained
*s*′	14	2.80	0.04	−0.07	−0.16 to 0.01	0.09	Maintained
Left ventricular diastolic function							
*E*	22	2.53	0.25	−0.40	−0.89 to 0.09	0.11	Maintained
*A*	20	3.98	0.25	−0.50	−1.00 to −0.01	**0.046**	Increased. There was an increase of ~ 0.50 cm·s^−1^ per year in controls relative to athletes
*E*/*A*	23	0.0001	0.004	0.00004	−0.01 to 0.01	0.99	Maintained
*e*′	14	0.41	0.06	−0.04	−0.17 to 0.09	0.52	Maintained
*a*′	13	1.03	0.04	−0.04	−0.12 to 0.04	0.31	Maintained

Bold values indicate statistical significance (*p* < 0.05)

*A* late diastolic mitral inflow velocity, *a*′ late diastolic tissue velocity, *CI* confidence interval, *E* early diastolic mitral inflow velocity, *e*′ early diastolic tissue velocity, *E/A* ratio of early to late mitral inflow velocity, *EF* ejection fraction, *FS* fractional shortening, *IVS* interventricular septal, *LVEDD* left ventricular end-diastolic diameter, *LVEDV* left ventricular end-diastolic volume, *LVM* left ventricular mass, *LVMi* left ventricular mass index, *PWT* posterior wall thickness, *RWT* relative wall thickness, *s*′ systolic tissue velocity, *SE* standard error

### Publication Bias

Egger’s regression revealed that LVMi was the only variable which demonstrated significant bias (Electronic Supplementary Material Table S2).

## Discussion

This first systematic pooling of controlled echocardiographic evidence of cardiac structure and function in aerobically (endurance) trained older athletes compared with age-matched untrained controls reveals that (1) endurance-trained older athletes have superior diastolic function compared with untrained counterparts, which is sustained regardless of advancing age; (2) mean LVM of older endurance athletes is greater (mean difference 72 g) than that of controls, as evidenced by greater wall thickness (mean ~ 1.2 mm) and chamber diameter (mean difference 3.7 mm); (3) despite modest differences in global systolic function between groups, older athletes have a larger SV (equivalent to mean difference ~ 1 L·min^−1^ greater cardiac output at rest) than controls, and this difference is sustained with chronological ageing.

### Left Ventricular Diastolic Function

Older athletes enjoy more favourable global diastolic function which is driven by a reduced reliance on late diastolic filling and evident in both haemodynamic and tissue assessment. Greater *E*/*A* in aerobic athletes both contrasts [12] and concurs [13] with previous meta-analyses in younger cohorts. The *E*/*A* difference between groups shown here (mean difference 0.18) compares well with that reported for younger individuals (mean difference 0.2) by Utomi et al. [13], suggesting that the difference in global diastolic function between aerobically trained athletes and untrained controls is age independent. This is endorsed by the finding of the present meta-regression. Collectively, this supports the tenet that global diastolic function assessed by mitral inflow is preserved in athletes (versus controls) from ~ 18 to 74 years of age. The improved global diastolic function mediated by reductions in *A*, without concomitant changes in *E*, accords with a large investigation of young, Olympic calibre athletes [60], highlighting a similar adaptation in athletes of all ages compared with untrained controls. Moreover, meta-regression analysis observed that the difference in *A* between athletes and controls *becomes more exaggerated with advancing age*, which suggests an incremental reliance on atrial contraction to support LV filling in untrained controls.

Greater *e*′ in older trained adults agrees with findings from Utomi et al. [13] in younger athletes. In our meta-analysis, athletes demonstrated lower *a*′, and when considered with the observed difference in early diastole, a greater *e*′/*a*′ was observed in athletes than in controls. These observations support the precept that continuation of a high volume of aerobic exercise into advanced age has pleiotropic effects on cardiac function.

### Cardiac Structure

Older athletes presented with larger LV wall thicknesses, absolute and relative LVMs, chamber diameter and LVEDV, which is comparable with findings from large-scale meta-analyses in younger athletes [12, 13]. This indicates preload dependent cardiac adaptation, widely considered as a normal cardiac manifestation from aerobic training [61], and these data suggest the maintenance of this phenomenon beyond 45 years. However, study-to-study variances in athlete and control blood pressures may have contributed towards the between-study heterogeneity observed, particularly for LVMi. Slower resting heart rates in athletes could also contribute to the increased LVEDD and LVEDV by lengthening the diastolic filling period. Furthermore, the meta-regression indicated no association between age and LVM, yet a significant, negative association with age and LVMi. The latter finding suggests that the differences in allometrically scaled LVM to BSA progressively decreased with advancing age, and supports a recent proposition that differences in LVMi between athletes and controls are dampened with advancing age [20]. The evidence that chronological age mediates LVMi in ageing athletes has not been convincing, with studies finding that younger, but not older athletes, have larger LVMi than age-matched controls [62] or older athletes do have a larger LVMi than controls yet this is to a lesser extent than younger athletes versus untrained controls [37, 63]. In contrast, others have shown a training effect with no interaction between age and training [29, 32, 49, 56]. Additionally, meta-regression showed the difference in LVEDD between athletes and controls decreases by a mean ~ 0.14 mm each year. Taking these findings together, we speculate that a gradual lowering of exercise intensity and/or training volume in older athletes [8], with a corresponding loss of volume overload, explains their inability to maintain physiological LVEDD and thus, ventricular mass, which is an established stimulus for physiological eccentric remodelling in those participating in isotonic exercise [64].

LAD was similar between athletes and controls, which contrasts with a prior meta-analysis showing larger LAD and volumes in younger athletes than in controls [17]. However, the results of this review may be considered preliminary given the relatively small number of studies available for the analysis of LA structure. Atrial morphology in older endurance athletes, relative to normal controls, requires further study and is of clinical interest given the ongoing debate concerning the potential interaction between atrial structural remodelling and the known larger incidence of atrial fibrillation in endurance athletes [65].

Similarly, RVEDD did not differ between older athletes and controls, which agrees with previous work in younger endurance athletes. Yet, two of the three studies in this meta-analysis observed larger RVEDD in athletes than in controls, highlighting a biventricular enlargement [30, 35] but significant heterogeneity between studies. This could be explained by age, as the participants in the studies showing RV dilation were younger than those in the study showing comparable diameter between athletes and controls [37]. Given the observed meta-regression of reduced mean difference LVEDD between athletes and controls with chronological age, it may be possible for a similar observation regarding the right-sided heart; however, more data are required before a sufficiently powered meta-regression can be conducted.

### Left Ventricular Systolic Function

Conventional measures of LV systolic function, EF and FS were comparable between athletes and controls, as observed in younger cohorts [12, 13], which indicates preservation of resting EF and FS in older athletes. SV was greater in athletes than in controls and comparable with the literature for younger athletes [13]. In the absence of changes to EF, greater SV in athletes is likely a reflection of larger LVEDV and LVEDD [66], which is notably preserved despite increasing age.

Speckle tracking-derived GLS is a more sensitive marker of LV systolic (dys)function than conventionally measured EF [67]. However, GLS appeared to be unaltered between older aerobically trained athletes and controls, which agrees with the findings of a recent systematic synthesis of data for younger athletes [15]. There is emerging evidence that GLS is reduced in disease states [68–70] and associated with poor cardiac outcomes [71]. Studies of younger [72–74] and older individuals [75] have reported small increases in GLS after aerobic training programmes, and these are considered to be positive adaptations of the athlete’s heart [76]. However, with only five studies included in this meta-analysis, the small volume of work in older athletes requires expansion before more accurate estimations can be made, especially considering recent documentation of preserved GLS in lifelong exercisers, albeit in a mixed sex cohort [77].

### Limitations

#### Available Studies

Within the present systematic search there was a single study of female athletes [78]. Due to this near absence of literature in ageing female athletes, the present findings should only be generalised to ageing men. Similarly, there were only two (potentially eligible) studies that examined resistance training (i.e. bodybuilding exercise) [35, 79] in ageing men, which precluded comparisons between the two most common exercise modes. In particular, more data are needed to fully characterise LA size in masters athletes, including diameter and volume, since varying degrees of lifelong training hours were associated with LA volume but not dimension [80]. The preponderance of studies of the LV and paucity of studies reporting on the RV limited our inclusion of RV indices, and therefore, a greater focus on the right side of the heart is warranted in older athletes. Future work should also include STE assessment of both left and right sides of the heart, to enable further insight into intrinsic cardiac mechanics.

Reporting of athlete training was inconsistent between studies, which prevented additional meta-regression analyses of training years, volume and intensity to further elucidate the between-study heterogeneity. Therefore, more robust documentation with quantitative means ± SDs is required before the influence of training regimes can be documented within a narrower age range, since cardiac structure and function have been reported to be exercise dose dependent [81]. Many studies did not state the specific sport performed by the athletes, or enrolled endurance athletes from different sports with varying magnitudes of static and dynamic loading. To allow subgroup analysis on the type of sport in future meta-analysis, we suggest studies report the sports of their athletes.

#### Present Meta-Analysis

This meta-analysis has notable limitations. Firstly, we were confined to analysing male participants. Further, we did not include other measures of body size-adjusted LV morphology (IVS, PWT, LVEDD). This was due to few data and inconsistent scaling within the literature. Of the included studies for LVMi, five used either height^2.7^ or fat-free mass to index LVM, which prevented separate meta-analysis and regression using these parameters for indexing other than BSA. It is noteworthy that two studies [34, 51] did not disclose the sex of participants. To resolve this, we employed an educated assumption of male participants because average LVEDD and LVEDV greatly exceeded the normal range in females [34]. This was further endorsed when average group LVMi data scaled to BSA were at the upper end of the range in men [23, 51] and vastly exceeded the expected range in females. We acknowledge that the data presented in this meta-analysis are derived from cross-sectional studies. However, there exists a paucity of prospective studies of sufficient duration to adequately quantify the influence of chronological age on the observed athlete–control differences, in addition to having relatively small sample sizes and mixed sexes. Indeed, 1 year of endurance training in older adults (> 65 years) did not alter LV stiffness and compliance [9], whereas recently, Howden et al. [82] observed reduced LV stiffness in middle-aged adults following 2 years of high-intensity training. While these data provide invaluable knowledge pertaining to the optimal stage of life to initiate exercise for offsetting the ageing process, the present findings document the benefit of exercise in those already trained across the age spectrum.

## Conclusions

Pooled information from controlled echocardiography studies demonstrates that older athletes have superior global diastolic function compared with controls because of a reduced reliance on atrial contraction to LV filling in mitral inflow velocity and both increased early and reduced late diastolic tissue velocities. Furthermore, older athletes have notable differences in cardiac structure (wall thickness, cavity size and LV volume) with greater relative and absolute LVM. Despite unremarkable differences in LV systolic function, SV is markedly greater in older athletes. Notably, the present data also identify that masters athletes maintain these functional effects during chronological ageing.

## Electronic supplementary material

Below is the link to the electronic supplementary material.

Supplementary material 1 (DOCX 13 kb)

Supplementary material 2 (DOCX 418 kb)
