# Supplementary material for: Aerobic Training Protects Cardiac Function During Advancing Age: A Meta-Analysis of Four Decades of Controlled Studies
Source: Sports Med. 2018 Oct 29;49(2):199–219. doi: 10.1007/s40279-018-1004-3 (PMC6513799; doi:10.1007/s40279-018-1004-3)
Supplement: Supplementary file 1 — Supplementary material 1 (DOCX 13 kb) [file 40279_2018_1004_MOESM1_ESM.docx]

**Electronic Supplementary Material Appendix S1**

Truncated terms, Boolean operators (AND/OR (all databases)) and associated exploded Medical Subject Headings (MeSH) terms (PubMed and MEDLINE) were incorporated into each individual search strand and then together for a combined search. PubMed search was as follows: echocardiography[MeSH Terms] OR echocardio*[Title/Abstract] OR speckle[Title/Abstract]; AND ventricular function[MeSH Terms] OR function*[Title/Abstract] OR structur*[Title/Abstract] OR strain[Title/Abstract] OR adaptation, physiological[MeSH Terms] AND exercise[MeSH Terms] OR resistance train*[Title/Abstract] OR aerobic train*[Title/Abstract] OR weight train*[Title/Abstract] OR interval training[Title/Abstract] OR aerobic[Title/Abstract] OR strength[Title/Abstract] OR athletes[Title/Abstract] AND aged[MeSH Terms] OR senior*[Title/Abstract] OR elderly[Title/Abstract] OR older[Title/Abstract] OR masters[Title/Abstract] OR veteran[Title/Abstract] NOT heart failure[MeSH Terms]. Reference lists from review articles and the authors own reference lists were screened for relevant records.
