# Supplementary material for: Aerobic Training Protects Cardiac Function During Advancing Age: A Meta-Analysis of Four Decades of Controlled Studies
Source: Sports Med. 2018 Oct 29;49(2):199–219. doi: 10.1007/s40279-018-1004-3 (PMC6513799; doi:10.1007/s40279-018-1004-3)
Supplement: Supplementary file 2 — Supplementary material 2 (DOCX 418 kb) [file 40279_2018_1004_MOESM2_ESM.docx]

Electronic Supplementary Material Table S1

| **No.** | **Characteristic** | **No** | **Yes** | **Max score** |
| --- | --- | --- | --- | --- |
| **1** | Is a power calculation reported? |  |  | **1** |
| **2** | Are inclusion/exclusion criteria stated relating to participant health? |  |  | **1** |
| ***Test - Controls*** | | | | |
| **3** | Are activity levels for the control group reported? If not, but participation in some sports is stated, are VO2max/peak data reported? |  |  | **1** |
| **4** | Is it clear that the control group are matched for age? (i.e stated or tabulated without statistical significance) |  |  | **1** |
| **5** | Are control group sex reported? |  |  | **1** |
| ***Test - Athletes*** | | | | |
| **6** | Are athletes training level clearly reported? (i.e elite, professional, national or international competition, amateur) for the whole group? If not, are VO2max/peak reported? |  |  | **1** |
| **7** | Are details available for training years/duration for the whole group? |  |  | **1** |
| **8** | Are details available for training volume/intensity? |  |  | **1** |
| **9** | Are athlete group sex reported? |  |  | **1** |
| **10** | Is the specific athlete sport reported? |  |  | **1** |
| ***Image acquisition*** | | | | |
| **11** | In general, are image acquisition techniques sufficiently detailed to allow replication for all variables of interest? (i.e views used, sample placement etc) If not, are measurement specific citations provided/guidelines acknowledged? |  |  | **1** |
| **12** | Is inter-observer or intra-observer variability stated? |  |  | **1** |
| **13** | Were investigators/assessors blinded for data analysis? |  |  | **1** |
| ***Measurement technique*** | | | | |
| **14** | Are professional guidelines observed/cited? |  |  | **1** |
| ***Reporting data*** | | | | |
| **15** | Is there clear indication of sample size for all variables of interest after analysis? (i.e ‘*n*’ reported or confirmation of satisfactory echo in all subjects) |  |  | **1** |
| **16** | Are data clearly and accurately presented (SD)? |  |  | **1** |
| **17** | Are absolute data values stated? |  |  | **1** |
| ***Total score*** | |  |  | **/17** |
|  |  |  |  |  |

Electronic Supplementary Material Table S2

| Measure | Number of Comparisons | Intercept | Two-tailed *P* Value |
| --- | --- | --- | --- |
| IVS | 18 | -0.85 | 0.35 |
| PWT | 18 | -1.33 | 0.12 |
| LVEDD | 23 | -1.92 | 0.09 |
| RWT | 11 | 2.27 | 0.23 |
| LVM | 10 | -3.74 | 0.16 |
| LVMi | 20 | 3.58 | **0.04** |
| LVEDV | 10 | 2.03 | 0.22 |
| LAD | 5 | 0.90 | 0.93 |
| RVEDD | 3 | 30.56 | 0.66 |
| EF | 15 | -0.79 | 0.68 |
| FS | 11 | -0.35 | 0.81 |
| SV | 7 | -0.71 | 0.65 |
| s’ | 14 | -0.30 | 0.88 |
| GLS | 5 | 4.92 | 0.29 |
| E | 22 | 0.31 | 0.78 |
| A | 20 | 0.18 | 0.89 |
| E/A | 23 | -0.46 | 0.54 |
| e’ | 14 | 1.90 | 0.48 |
| a’ | 13 | 0.17 | 0.93 |
| e’/a’ | 7 | 5.01 | 0.14 |
| E/e’ | 8 | 1.45 | 0.51 |

A, late diastolic mitral inflow velocity; a’, late diastolic tissue velocity; E, early diastolic mitral inflow velocity; e’, early diastolic tissue velocity; e’/a’, ratio of early-to-late diastolic tissue velocity; E/A, ratio of early-to-late mitral inflow velocity; E/e’, ratio of early diastolic mitral inflow velocity-to-early diastolic tissue velocity; EF, ejection fraction; FS, fractional shortening; GLS, global longitudinal strain; IVS, interventricular septal thickness; LAD, left atrial diameter; LVEDD, left ventricular end-diastolic diameter; LVM, left ventricular mass; LVMi, left ventricular mass index; PWT, posterior wall thickness; RVEDD, right ventricular end-diastolic diameter; RWT, relative wall thickness; s’, systolic tissue velocity; and SV, stroke volume. Bold values indicate statistical significance.

# Electronic Supplementary Material Figure S1

#

Forest plot showing meta-analysis of athlete-control differences for interventricular septal thickness (IVS) represented as the differences in means (mm). CI, confidence intervals; *, represents undefined sport but are described as either endurance or aerobic athletes. Forest Plot Symbols; Closed square, study effect size; the size of symbol and the CIs represent study weight and precision, respectively in the meta-analysis; closed diamond, overall summary effect.

# Electronic Supplementary Material Figure S2

Forest plot showing meta-analysis of athlete-control differences for posterior wall thickness (PWT) represented as the differences in means (mm). CI, confidence intervals; *, represents undefined sport but are described as either endurance or aerobic athletes. Forest Plot Symbols; Closed square, study effect size; the size of symbol and the CIs represent study weight and precision, respectively in the meta-analysis; closed diamond, overall summary effect.

# Electronic Supplementary Material Figure S3

Forest plot showing meta-analysis of athlete-control differences for left ventricular end-diastolic diameter (LVEDD) represented as the differences in means (mm). CI, confidence intervals; *, represents undefined sport but are described as either endurance or aerobic athletes. Forest Plot Symbols; Closed square, study effect size; the size of symbol and the CIs represent study weight and precision, respectively in the meta-analysis; closed diamond, overall summary effect.

# Electronic Supplementary Material Figure S4

Forest plot showing meta-analysis of athlete-control differences for left ventricular end-diastolic volume (LVEDV) represented as the differences in means (mL). CI, confidence intervals; *, represents undefined sport but are described as either endurance or aerobic athletes. Forest Plot Symbols; Closed square, study effect size; the size of symbol and the CIs represent study weight and precision, respectively in the meta-analysis; closed diamond, overall summary effect.

# Electronic Supplementary Material Figure S5

Forest plot showing meta-analysis of athlete-control differences for relative wall thickness (RWT) represented as the differences in means. CI, confidence intervals; *, represents undefined sport but are described as either endurance or aerobic athletes; 1, and 2 denote multiple athlete-control comparisons from the same study. Forest Plot Symbols; Closed square, study effect size; the size of symbol and the CIs represent study weight and precision, respectively in the meta-analysis; closed diamond, overall summary effect.

# Electronic Supplementary Material Figure S6

Forest plot showing meta-analysis of athlete-control differences for left ventricular mass (LVM) represented as the differences in means (g). CI, confidence intervals; *, represents undefined sport but are described as either endurance or aerobic athletes. Forest Plot Symbols; Closed square, study effect size; the size of symbol and the CIs represent study weight and precision, respectively in the meta-analysis; closed diamond, overall summary effect.

# Electronic Supplementary Material Figure S7

Forest plot showing meta-analysis of athlete-control differences for left ventricular mass index (LVMi) represented as the standardised differences in means (*d*). CI, confidence intervals; *, represents undefined sport but are described as either endurance or aerobic athletes. Forest Plot Symbols; Closed square, study effect size; the size of symbol and the CIs represent study weight and precision, respectively in the meta-analysis; closed diamond, overall summary effect.

# Electronic Supplementary Material Figure S8

Forest plot showing meta-analysis of athlete-control differences for left atrial diameter (LAD) represented as the differences in means (mm). CI, confidence intervals; *, represents undefined sport but are described as either endurance or aerobic athletes. Forest Plot Symbols; Closed square, study effect size; the size of symbol and the CIs represent study weight and precision, respectively in the meta-analysis; closed diamond, overall summary effect.

# Electronic Supplementary Material Figure S9

Forest plot showing meta-analysis of athlete-control differences for right ventricular end-diastolic diameter (RVEDD) represented as the differences in means (mm). CI, confidence intervals; *, represents undefined sport but are described as either endurance or aerobic athletes. Forest Plot Symbols; Closed square, study effect size; the size of symbol and the CIs represent study weight and precision, respectively in the meta-analysis; closed diamond, overall summary effect.

# Electronic Supplementary Material Figure S10

Forest plot showing meta-analysis of athlete-control differences for ejection fraction (EF) represented as the differences in means (%). CI, confidence intervals; *, represents undefined sport but are described as either endurance or aerobic athletes. Forest Plot Symbols; Closed square, study effect size; the size of symbol and the CIs represent study weight and precision, respectively in the meta-analysis; closed diamond, overall summary effect.

# Electronic Supplementary Material Figure S11

Forest plot showing meta-analysis of athlete-control differences for fractional shortening (FS) represented as the differences in means (%). CI, confidence intervals; *, represents undefined sport but are described as either endurance or aerobic athletes. Forest Plot Symbols; Closed square, study effect size; the size of symbol and the CIs represent study weight and precision, respectively in the meta-analysis; closed diamond, overall summary effect.

# Electronic Supplementary Material Figure S12

Forest plot showing meta-analysis of athlete-control differences for stroke volume (SV) represented as the differences in means (mL). CI, confidence intervals; *, represents undefined sport but are described as either endurance or aerobic athletes. Forest Plot Symbols; Closed square, study effect size; the size of symbol and the CIs represent study weight and precision, respectively in the meta-analysis; closed diamond, overall summary effect.

# Electronic Supplementary Material Figure S13

Forest plot showing meta-analysis of athlete-control differences for systolic tissue velocity (s’) represented as the differences in means (cm.s^-1^). CI, confidence intervals; *, represents undefined sport but are described as either endurance or aerobic athletes. Forest Plot Symbols; Closed square, study effect size; the size of symbol and the CIs represent study weight and precision, respectively in the meta-analysis; closed diamond, overall summary effect.

# Electronic Supplementary Material Figure S14

#

Forest plot showing meta-analysis of athlete-control differences for global longitudinal strain (GLS) represented as the differences in means (%). CI, confidence intervals. Forest Plot Symbols; Closed square, study effect size; the size of symbol and the CIs represent study weight and precision, respectively in the meta-analysis; closed diamond, overall summary effect.

#

# Electronic Supplementary Material Figure S15

Forest plot showing meta-analysis of athlete-control differences for early mitral inflow velocity (E) represented as the differences in means (cm.s^-1^). CI, confidence intervals; *, represents undefined sport but are described as either endurance or aerobic athletes. Forest Plot Symbols; Closed square, study effect size; the size of symbol and the CIs represent study weight and precision, respectively in the meta-analysis; closed diamond, overall summary effect.

# Electronic Supplementary Material Figure S16

Forest plot showing meta-analysis of athlete-control differences for late mitral inflow velocity (A) represented as the differences in means (cm.s^-1^). CI, confidence intervals; *, represents undefined sport but are described as either endurance or aerobic athletes. Forest Plot Symbols; Closed square, study effect size; the size of symbol and the CIs represent study weight and precision, respectively in the meta-analysis; closed diamond, overall summary effect.

# Electronic Supplementary Material Figure S17

Forest plot showing meta-analysis of athlete-control differences for the ratio of early-to-late mitral inflow velocity (E/A) represented as the differences in means. CI, confidence intervals; *, represents undefined sport but are described as either endurance or aerobic athletes; 1, and 2 denote multiple athlete-control comparisons from the same study. Forest Plot Symbols; Closed square, study effect size; the size of symbol and the CIs represent study weight and precision, respectively in the meta-analysis; closed diamond, overall summary effect.

# Electronic Supplementary Material Figure S18

Forest plot showing meta-analysis of athlete-control differences for early diastolic tissue velocity (e’) represented as the differences in means (cm.s^-1^). CI, confidence intervals; *, represents undefined sport but are described as either endurance or aerobic athletes. Forest Plot Symbols; Closed square, study effect size; the size of symbol and the CIs represent study weight and precision, respectively in the meta-analysis; closed diamond, overall summary effect.

# Electronic Supplementary Material Figure S19

Forest plot showing meta-analysis of athlete-control differences for late diastolic tissue velocity (a’) represented as the differences in means (cm.s^-1^). CI, confidence intervals; *, represents undefined sport but are described as either endurance or aerobic athletes. Forest Plot Symbols; Closed square, study effect size; the size of symbol and the CIs represent study weight and precision, respectively in the meta-analysis; closed diamond, overall summary effect.

# Electronic Supplementary Material Figure S20

Forest plot showing meta-analysis of athlete-control differences for early-to-late diastolic tissue velocity (e’/a’) represented as the differences in means. CI, confidence intervals; *, represents undefined sport but are described as either endurance or aerobic athletes. Forest Plot Symbols; Closed square, study effect size; the size of symbol and the CIs represent study weight and precision, respectively in the meta-analysis; closed diamond, overall summary effect.

# Electronic Supplementary Material Figure S21

Forest plot showing meta-analysis of athlete-control differences for the ratio of early mitral inflow velocity-to-early diastolic tissue velocity (E/e’) represented as the differences in means. CI, confidence intervals; *, represents undefined sport but are described as either endurance or aerobic athletes. Forest Plot Symbols; Closed square, study effect size; the size of symbol and the CIs represent study weight and precision, respectively in the meta-analysis; closed diamond, overall summary effect.

# Electronic Supplementary Material Figure S22

#

Meta-regression of athlete – control difference in means with age for interventricular septal thickness. Each circle represents an individual study and the size of the circle reflects the study weight.

# Electronic Supplementary Material Figure S23

Meta-regression of athlete – control difference in means with age for posterior wall thickness. Each circle represents an individual study and the size of the circle reflects the study weight.

# Electronic Supplementary Material Figure S24

Meta-regression of athlete – control difference in means with age for left ventricular end-diastolic diameter. Each circle represents an individual study and the size of the circle reflects the study weight.

# Electronic Supplementary Material Figure S25

#
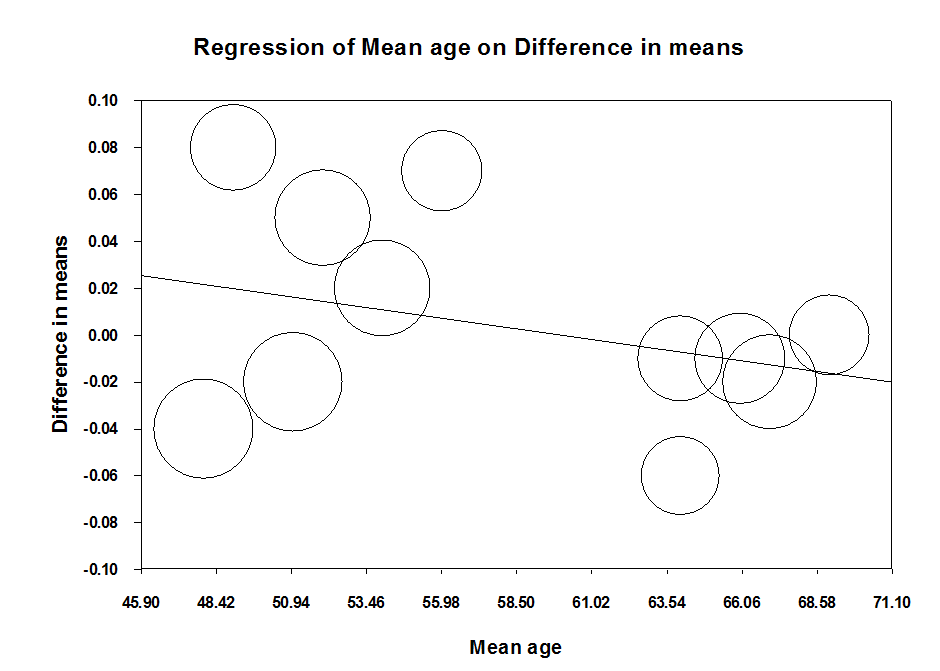


Meta-regression of athlete – control difference in means with age for relative wall thickness. Each circle represents an individual study and the size of the circle reflects the study weight.

# Electronic Supplementary Material Figure S26

Meta-regression of athlete – control difference in means with age for left ventricular end-diastolic volume. Each circle represents an individual study and the size of the circle reflects the study weight.

# Electronic Supplementary Material Figure S27

Meta-regression of athlete – control difference in means with age for left ventricular mass. Each circle represents an individual study and the size of the circle reflects the study weight.

# Electronic Supplementary Material Figure S28

Meta-regression of athlete – control standardized difference in means with age for left ventricular mass index. Each circle represents an individual study and the size of the circle reflects the study weight.

# Electronic Supplementary Material Figure S29

#

Meta-regression of athlete – control difference in means with age for ejection fraction. Each circle represents an individual study and the size of the circle reflects the study weight.

# Electronic Supplementary Material Figure S30

#
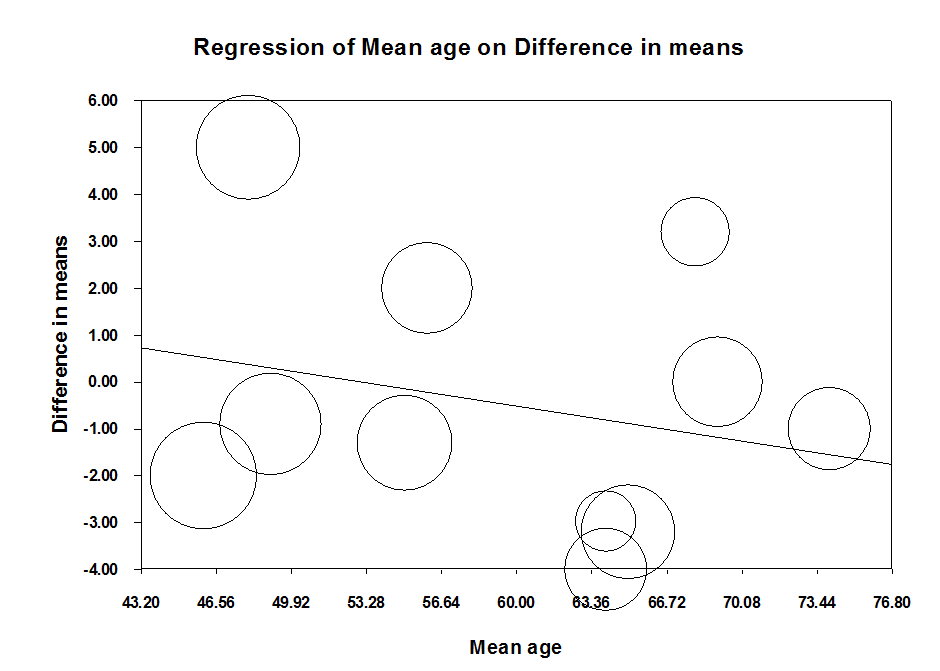


Meta-regression of athlete – control difference in means with age for fractional shortening. Each circle represents an individual study and the size of the circle reflects the study weight.

# Electronic Supplementary Material Figure S31

Meta-regression of athlete – control difference in means with age for systolic tissue velocity. Each circle represents an individual study and the size of the circle reflects the study weight.

# Electronic Supplementary Material Figure S32

Meta-regression of athlete – control difference in means with age for early mitral inflow velocity. Each circle represents an individual study and the size of the circle reflects the study weight.

# Electronic Supplementary Material Figure S33

Meta-regression of athlete – control difference in means with age for late mitral inflow velocity. Each circle represents an individual study and the size of the circle reflects the study weight.

# Electronic Supplementary Material Figure S34

Meta-regression of athlete – control difference in means with age for the ratio of early-to-late mitral inflow velocity. Each circle represents an individual study and the size of the circle reflects the study weight.

# Electronic Supplementary Material Figure S35

#

Meta-regression of athlete – control difference in means with age for early diastolic tissue velocity. Each circle represents an individual study and the size of the circle reflects the study weight.

# Electronic Supplementary Material Figure S36

Meta-regression of athlete – control difference in means with age for late diastolic tissue velocity. Each circle represents an individual study and the size of the circle reflects the study weight.

**References**

1. Baldi JC, McFarlane K, Oxenham HC, Whalley GA, Walsh HJ, Doughty RN. Left ventricular diastolic filling and systolic function of young and older trained and untrained men. J Appl Physiol Bethesda Md 1985. 2003;95:2570–5.

2. Bohm P, Schneider G, Linneweber L, Rentzsch A, Krämer N, Abdul-Khaliq H, et al. Right and left ventricular function and mass in male elite master athletes: A controlled contrast enhanced CMR study. Circulation. 2016;CIRCULATIONAHA.115.020975.

3. Bouvier F, Saltin B, Nejat M, Jensen-Urstad M. Left ventricular function and perfusion in elderly endurance athletes. Med Sci Sports Exerc. 2001;33:735–40.

4. Carrick-Ranson G, Doughty RN, Whalley GA, Walsh HJ, Gamble GD, Baldi JC. The larger exercise stroke volume in endurance-trained men does not result from increased left ventricular early or late inflow or tissue velocities. Acta Physiol Oxf Engl. 2012;205:520–31.

5. Child JS, Barnard RJ, Taw RL. Cardiac hypertrophy and function in master endurance runners and sprinters. J Appl Physiol. 1984;57:176–81.

6. Cottini E, Giacone G, Cosentino M, Cirino A, Rando G, Vintaloro G. Evaluation of left ventricular diastolic function by pulmonary venous and mitral flow velocity patterns in endurance veteran athletes. Arch Gerontol Geriatr. 1996;22 Suppl 1:179–86.

7. D’Andrea A, Caso P, Scarafile R, Salerno G, De Corato G, Mita C, et al. Biventricular myocardial adaptation to different training protocols in competitive master athletes. Int J Cardiol. 2007;115:342–9.

8. Di Bello V, Lattanzi F, Picano E, Talarico L, Caputo MT, Di Muro C, et al. Left ventricular performance and ultrasonic myocardial quantitative reflectivity in endurance senior athletes: an echocardiographic study. Eur Heart J. 1993;14:358–63.

9. Donal E, Rozoy T, Kervio G, Schnell F, Mabo P, Carré F. Comparison of the heart function adaptation in trained and sedentary men after 50 and before 35 years of age. Am J Cardiol. 2011;108:1029–37.

10. Fleg JL, Shapiro EP, O’Connor F, Taube J, Goldberg AP, Lakatta EG. Left ventricular diastolic filling performance in older male athletes. Jama. 1995;273:1371–5.

11. Galetta F, Franzoni F, Santoro G, Prattichizzo F, Femia FR, Pastine F, et al. QT dispersion in elderly athletes with left ventricular hypertrophy. Int J Sports Med. 2003;24:233–7.

12. Galetta F, Franzoni F, Femia FR, Bartolomucci F, Carpi A, Santoro G. Left ventricular diastolic function and carotid artery wall in elderly athletes and sedentary controls. Biomed Pharmacother Biomedecine Pharmacother. 2004;58:437–42.

13. Gates PE, Tanaka H, Graves J, Seals DR. Left ventricular structure and diastolic function with human ageing. Relation to habitual exercise and arterial stiffness. Eur Heart J. 2003;24:2213–20.

14. Giada F, Bertaglia E, De Piccoli B, Franceschi M, Sartori F, Raviele A, et al. Cardiovascular adaptations to endurance training and detraining in young and older athletes. Int J Cardiol. 1998;65:149–55.

15. Grace F, Herbert P, Elliott AD, Richards J, Beaumont A, Sculthorpe NF. High intensity interval training (HIIT) improves resting blood pressure, metabolic (MET) capacity and heart rate reserve without compromising cardiac function in sedentary aging men. Exp Gerontol. 2017;

16. Jungblut PR, Osborne JA, Quigg RJ, McNeal MA, Clauser J, Muster AJ, et al. Echocardiographic Doppler evaluation of left ventricular diastolic filling in older, highly trained male endurance athletes. Echocardiogr Mt Kisco N. 2000;17:7–16.

17. Kozàkovà M, Galetta F, Gregorini L, Bigalli G, Franzoni F, Giusti C, et al. Coronary vasodilator capacity and epicardial vessel remodeling in physiological and hypertensive hypertrophy. Hypertens Dallas Tex 1979. 2000;36:343–9.

18. Lee LS, Mariani JA, Sasson Z, Goodman JM. Exercise with a twist: left ventricular twist and recoil in healthy young and middle-aged men, and middle-aged endurance-trained men. J Am Soc Echocardiogr Off Publ Am Soc Echocardiogr. 2012;25:986–93.

19. Lindsay MM, Dunn FG. Biochemical evidence of myocardial fibrosis in veteran endurance athletes. Br J Sports Med. 2007;41:447–52.

20. Maessen MF, Eijsvogels TM, Stevens G, van Dijk AP, Hopman MT. Benefits of lifelong exercise training on left ventricular function after myocardial infarction. Eur J Prev Cardiol. 2017;2047487317728765.

21. Matelot D, Schnell F, Kervio G, Ridard C, Thillaye du Boullay N, Wilson M, et al. Cardiovascular benefits of endurance training in seniors: 40 is not too late to start. Int J Sports Med. 2016;37:625–32.

22. Maufrais C, Doucende G, Rupp T, Dauzat M, Obert P, Nottin S, et al. Left ventricles of aging athletes: better untwisters but not more relaxed during exercise. Clin Res Cardiol. 2017;1–9.

23. Maufrais C, Schuster I, Doucende G, Vitiello D, Rupp T, Dauzat M, et al. Endurance training minimizes age-related changes of left ventricular twist-untwist mechanics. J Am Soc Echocardiogr Off Publ Am Soc Echocardiogr. 2014;27:1208–15.

24. Miki T, Yokota Y, Seo T, Yokoyama M. Echocardiographic findings in 104 professional cyclists with follow-up study. Am Heart J. 1994;127:898–905.

25. Molmen HE, Wisloff U, Aamot IL, Stoylen A, Ingul CB. Aerobic interval training compensates age related decline in cardiac function. Scand Cardiovasc J SCJ. 2012;46:163–71.

26. Nishimura T, Yamada Y, Kawai C. Echocardiographic evaluation of long-term effects of exercise on left ventricular hypertrophy and function in professional bicyclists. Circulation. 1980;61:832–40.

27. Northcote RJ, McKillop G, Todd IC, Canning GP. The effect of habitual sustained endurance exercise on cardiac structure and function. Eur Heart J. 1990;11:17–22.

28. Nottin S, Nguyen L-D, Terbah M, Obert P. Long-term endurance training does not prevent the age-related decrease in left ventricular relaxation properties. Acta Physiol Scand. 2004;181:209–15.

29. Olsen RH, Couppé C, Dall CH, Monk-Hansen T, Mikkelsen UR, Karlsen A, et al. Age-related decline in mitral peak diastolic velocities is unaffected in well-trained runners. Scand Cardiovasc J SCJ. 2015;49:183–92.

30. Sagiv M, Goldhammer E, Ben-Sira D, Amir R. What maintains energy supply at peak aerobic exercise in trained and untrained older men? Gerontology. 2007;53:357–61.

31. Schmidt JF, Andersen TR, Andersen LJ, Randers MB, Hornstrup T, Hansen PR, et al. Cardiovascular function is better in veteran football players than age-matched untrained elderly healthy men. Scand J Med Sci Sports. 2015;25:61–9.

32. Seals DR, Hagberg JM, Spina RJ, Rogers MA, Schechtman KB, Ehsani AA. Enhanced left ventricular performance in endurance trained older men. Circulation. 1994;89:198–205.
